# Site‐Engineered Corundum‐Structured Aesthetic Composites for Alleviating Heat Accumulation to Mitigate Urban Heat Islands

**DOI:** 10.1002/smll.202513911

**Published:** 2026-03-15

**Authors:** G.P. Darshan, Akshay Arjun, Subhendu Mishra, Abhishek Kumar Singh, Alberto Vomiero, Elisa Moretti

**Affiliations:** ^1^ Physics and Astronomy Department University of Padova Padova Italy; ^2^ Department of Physics Faculty of Natural Sciences Ramaiah Technology Campus M S Ramaiah University of Applied Sciences Bengaluru India; ^3^ Department of Physics RNS Institute of Technology Bengaluru Karnataka India; ^4^ Materials Research Centre Indian Institute of Science Bengaluru India; ^5^ Department of Molecular Sciences and Nanosystems Ca’ Foscari University of Venice Venezia Italy; ^6^ Division of Materials Science Department of Engineering Sciences and Mathematics Luleå University of Technology Luleå Sweden

**Keywords:** Chromophores, NIR reflectance, Photometric parameters, Urban heat islands

## Abstract

Urban heat islands have become a global issue, as they severely impact several key factors, including elevated energy consumption, increased air pollution and greenhouse gas emissions, compromised human health and comfort, and reduced water quality. This escalating issue has become a concern and requires strategic technology deployment to address the threat. In this view, the present work deals with the synthesis of corundum‐structured and octahedral site‐engineered Mg_3−_ *_x_* A*_x_* TeO_6_ (A = Fe, and Co; *x* = 0.05–0.25 wt.%) pigments with brilliant colors via a solution combustion route. The designed corundum‐structured Mg_3−_ *_x_* A*_x_* TeO_6_ pigments exhibit an isostructural geometry with a centrosymmetric space group ($\bar{R_{3}}$). The prepared pigments exhibit aesthetically pleasing and vibrant brown and purple hues through the engineering of site‐specific (Mg/Fe/Co)O_6_ octahedral positions within the corundum‐structured Mg_3_TeO_6_ lattice. The colors of the pigments were manipulated (i.e., from absolute white to brown and purple colors) by integrating specific concentrations of transition‐metal chromophores. The prepared best‐performing Mg_2.85_Fe_0.15_TeO_6_ and Mg_2.85_Co_0.15_TeO_6_ pigments showcase their capability of delivering exceptional average reflectance properties in the near‐infrared region (800–2500 nm) of ∼86%. The integration of transition‐metal ions into corundum‐structured Mg_3_TeO_6_ could effectively modulate its electronic structure through hybridization between O(p) and Fe(d)/Co(d) states, thereby enhancing the near‐infrared reflectance of the pigments. The prepared pigments delivered a low thermal conductivity of about 0.06 and 0.08 W/m.K, which signifies their candidacy in cooling systems over traditional roofing materials. The cooling pigments demonstrated their stability against acid/alkali treatment, photo‐resistivity, and thermal stability. The deficient electricity demand as compared to bare cement and TiO_2_‐mica pearlescent pigment coating was evidently witnessed when the prepared pigments were used as cool coatings in an energy simulation. All clarifies the suitability of pigments in real‐time implementation.

## Graphical abstract

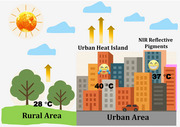

## 1 Introduction

**FIGURE 1:**
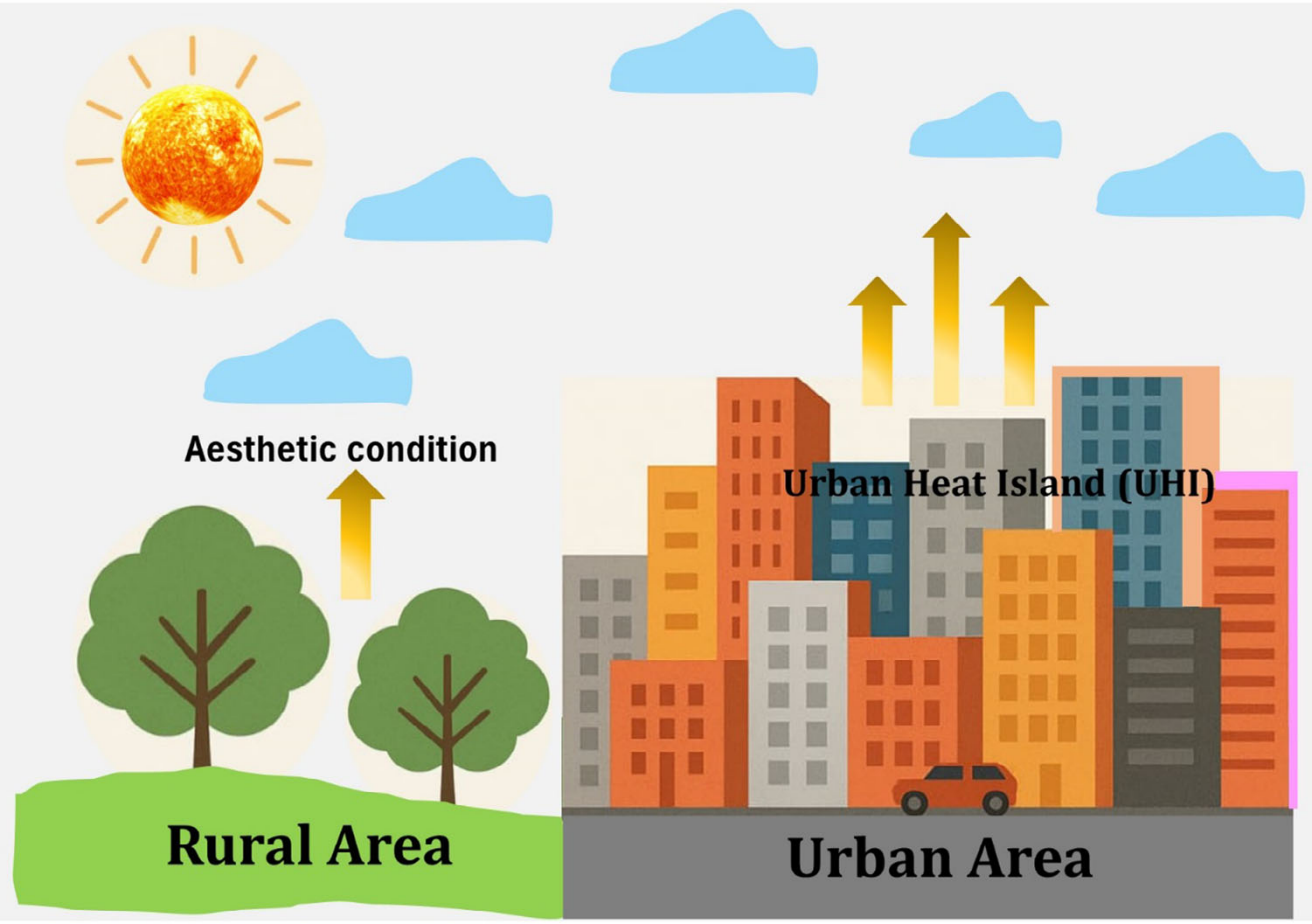
Schematic illustration demonstrating the possible UHI.

Over the past few decades, the “urban heat islands” (UHIs) effect has become a significant issue for environmental sustainability and public health [1, 2]. Heat islands refer to urban regions that have elevated temperatures compared to surrounding areas [3]. Heat islands develop due to various factors, including the loss of natural landscapes in cities, the properties of urban materials, the layout of urban areas, heat produced by human activities, as well as weather and geographical conditions, among others [4, 5]. The developed UHIs severely impacted several factors, including elevated energy consumption, increased air pollutants and greenhouse gas emissions, compromising human health and comfort, and lowering water quality [6]. The UHIs increase electricity demand for air‐conditioning, with a 1% to 9% rise in electricity needed for each 2° F increase in temperature. The resulting higher electricity costs often lead utility firms to rely on fossil fuel power plants, which increase greenhouse gas emissions and contribute to climate change. In addition to being detrimental to human health, these pollutants lead to serious air quality issues, like acid rain, fine particulate matter, and smog. Moreover, the generated hot weather from the UHIs can lead to more heat‐related illnesses and deaths. A survey by the *Centers for Disease Control and Prevention* found that the United States of America (USA) recorded around 702 heat‐related deaths each year from 2004–2018 [7]. The schematic illustration demonstrating the possible UHIs was depicted in Figure 1.

Currently, there are numerous methods available for mitigating the severity of the UHIs effect, including enhancing trees and vegetation, implementing green roofs, constructing cool roofs, utilizing cool pavements, adjusting to heat, and adhering to smart growth principles [8]. Among mitigating solutions, cool roofs have been considered an impactful strategy due to their advantages beyond reducing UHIs, such as: (i) reduced energy use – cool roof's solar reflectance can lower peak cooling demand in air‐conditioned buildings by 11–27%, (ii) decreased air pollution and greenhouse gas emissions – cool roofs minimize greenhouse gas emissions and subsequent air pollution by reducing energy consumption, and (iii) better human comfort and health – when installed in whole city, cool roof would mitigate 18% of heat‐related deaths linked to the UHI effect [9, 10, 11]. Nevertheless, near‐infrared (NIR) radiation is considered to be the main heating source to develop UHI, since 52% of solar radiation falls in the NIR region. In this regard, effective coating of NIR reflective pigments on the buildings, roofs, and roads in the urban region is a strategic method to alleviate the UHI effect [12]. To date, several attempts have been made by researchers to design aesthetically colored and NIR‐reflective pigments. As witnessed from Table S1, several pigments are synthesized by using rare earth (RE) elements and comprise toxic elements, such as Cr, Hg, As, and Cd, which are expensive and can lead to environmental contamination [13, 14]. However, white pigments like TiO_2_ exhibit high solar NIR reflectance but are susceptible to staining and can cause light pollution, such as glare. In this regard, research initiatives strive to enhance advantages while reducing drawbacks by focusing on eco‐friendly pigments without toxic elements. Economic aspects must also be taken into account, especially the elevated cost and limited availability of RE elements, which are crucial for advanced applications and should consequently be avoided.

In search of low‐cost and non‐toxic aesthetic pigments with high NIR‐reflectance, corundum‐structured materials belong to the gemstone family, which includes brilliant red rubies and blue sapphires [15]. The representative examples of corundum structure include α‐Al_2_O_3_, which belongs to a hexagonal close‐packed array of systems in which two‐thirds of the octahedral sites are occupied by Al^3+^ ions. The doping of Ti^4+^ and Fe^2+^ chromophore ions in the octahedral sites within the corundum structure results in blue sapphires as a consequence of intervalence charge transfer among the chromophores [16]. However, the replacement of Al^3+^ ions in the octahedral sites by Cr^3+^ ions leads to deep red rubies. With these advantages, the numerous corundum‐structured materials as a host for colored compounds have been extensively reported, but the study related to their NIR‐reflectance property was completely ignored [15, 17]. Among several corundum‐structured materials, Mg_3_TeO_6_ is an isostructural material that exhibits structural robustness, tunable bandgaps, and stability. The Mg_3_TeO_6_ has the centrosymmetric space group $\bar{R_{3}}$ with one distinct octahedral site for the Mg^2+^ ion. The substitution of transition‐metal (TM) ions in unusual and distorted coordination geometries of Mg_3_TeO_6_ (octahedral site) gains particular interest in view of the development of new vividly colored pigments. In addition, Mg_3_TeO_6_ exhibit wide bandgap (∼3.5 eV) semiconducting material [18], which prevents the absorption of lower‐energy NIR photons, since the photons in the NIR spectrum do not have enough energy to bridge the wide bandgap of these materials, and therefore makes it a promising candidate for the design of inorganic pigments with tunable NIR optical properties. Thus, there is an interest in rationally designing NIR reflective pigments through substituting TM ions in unusual and distorted coordination geometries.

Here, for the first time, we have investigated the TM ions‐doped corundum‐structured Mg_3−_ *_x_* A*_x_* TeO_6_ (A = Fe and Co; *x* = 0.05‐0.25 wt.%) pigments with brilliant colors and exceptional NIR‐reflectance properties were synthesized by the solution combustion (SC) route. The prepared corundum‐structured Mg_3−_ *_x_* A*_x_* TeO_6_ pigment exhibits isostructural geometry with a centrosymmetric space group ($\bar{R_{3}}$) and octahedral coordination environment for TM chromophores, which paves the way for exploration of different colors. The prepared pigments exhibit aesthetic and brilliant brown and purple colors via site‐specific integration of TM ions i.e., (Mg/Fe/Co)O_6_ octahedral position in the corundum‐structured Mg_3_TeO_6_ lattice. The best‐performing Mg_3−_ *_x_* Fe*_x_* TeO_6_ and Mg_3−_ *_x_* Co*_x_* TeO_6_ (*x* = 0.15 wt.%) pigments deliver exceptional average reflectance in the NIR region (800–2500 nm) of ∼ 86%. The photometric properties of the pigments showcase the obvious color tuning (i.e., from absolute white to brown and purple colors), which can be controlled by manipulating the concentration of dopant TM ions. In addition, pigments show excellent stability against various factors, such as chemicals, light irradiation, and temperature. The aforementioned results demonstrated their envisaged capabilities in mitigating UHIs.

## 2 Results and Discussion

**FIGURE 2:**
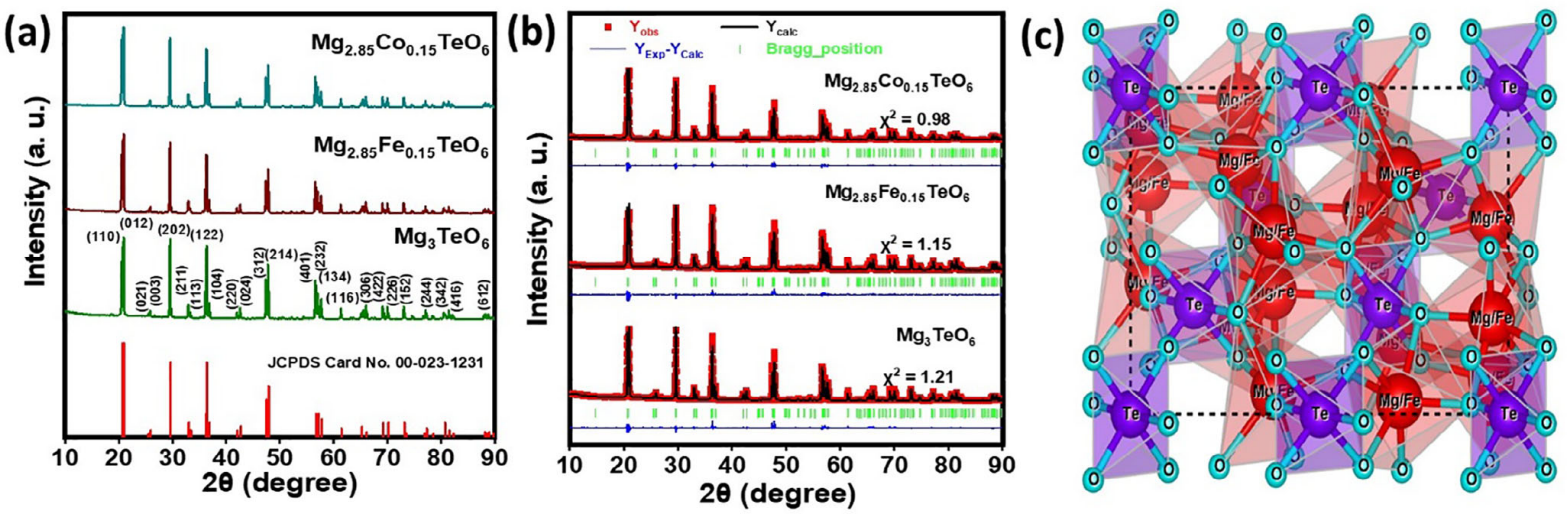
(a) XRD patterns, (b) Rietveld refinement of the pristine, Mg_2.85_Fe_0.15_TeO_6_, and Mg_2.85_Co_0.15_TeO_6_ pigments, and (c) Crystal structure of the prepared pigments.

The site‐engineered aesthetic Mg_3−_ *_x_* A*_x_* TeO_6_ (A = Fe and Co; *x* = 0.05–0.25 wt.%) pigments were synthesized via the SC route. The oxidizers and fuel are taken in a 1:1 ratio and kept in a muffle furnace maintained at 550°C for 10 min. The added fuel causes rapid disintegration of the oxidizers, releasing gases with a substantial amount of heat. The combustion results in a fluffy powder with portholes. The significant heat released during combustion plays a crucial role in forming the highly crystalline structure of the synthesized pigment. The obtained powder was finely ground, calcined at 700°C for 3 h, and subsequently used for further characterizations. X‐ray diffraction (XRD) patterns of the prepared pristine and Mg_3−_ *_x_* A*_x_* TeO_6_ (A = Fe and Co; *x* = 0.05–0.25 wt.%) pigments were displayed in Figure 2a and Figure S1 and S2. The distinct and intense diffraction peaks confirmed the hexagonal crystal structure with a centrosymmetric space group $\bar{R_{3}}$ [17]. All the diffraction peaks were in good agreement with the standard Mg_3_TeO_6_ (JCPDS card no. 00‐023‐1231). The XRD profiles do not show any irrelevant peaks related to impurities or dopant ions, which demonstrates that the dopant TM ions have been successfully incorporated into the Mg_3_TeO_6_ lattice by displacing the Mg^2+^ ion without generating any other phases. To confirm the effective substitution of TM ions in the lattice site, the ionic radii acceptable percentage difference (Δr) between the host cation Mg^2+^ (ionic radius = 0.72 Å in VI coordination) against dopant ions Co^2+^ (ionic radius = 0.65 Å in VI coordination), and Fe^2+^ (ionic radius = 0.61 Å in VI coordination) was estimated using the formula, as described in the literature [19]. The Δr values between Mg^2+^ – Fe^2+^, and Mg^2+^ – Co^2+^ were calculated and found to be 15.27%, and 9.72%, respectively. The estimated Δr values were in lieu within a standard value of acceptance (i.e., < 30%). Thanks to the well‐accepted ionic difference between the host Mg^2+^ ion and TM ions, the prepared pigments have a pure single hexagonal structural phase.

The detailed crystal structure visualization was analyzed via Rietveld refinements of the pristine, Mg_2.85_Fe_0.15_TeO_6_, and Mg_2.85_Co_0.15_TeO_6_ pigments. The refined results (shown in Figure 2b) indicated a robust correlation between the experimentally observed and calculated profiles. Furthermore, refined data analysis confirms the formation of hexagonal structure‐type pigments with a centrosymmetric space group $\bar{R_{3}}$. The various structural and reliability parameters elucidated through Rietveld's refinement of the pigments were summarized in Table S2. The fitting parameters (R_p_, R_wp_, and χ^2^) show a good agreement between the refined and observed XRD patterns. No significant changes in the lattice parameters or cell volume after substitution of dopants to the host lattice were witnessed, which is ascribed to the favorable ionic radii of Mg^2+^ (0.72 Å) and dopant ions (i.e., Co^2+^ (0.65 Å), and Fe^2+^ (0.61 Å in VI coordination). The possible lattice strain that arises following the successful incorporation of suitable dopants influences the electronic band structure of the Mg_2.85_Fe_0.15_TeO_6_ and Mg_2.85_Co_0.15_TeO_6_ pigments (witnessed from density of states (DOS)), which directly affects their reflection properties. However, the calculated structural parameters were employed to predict the crystal structure and surrounding atomic environment of the prepared pigments using the VESTA program. Figure 2c shows the hexagonal crystal structure of the prepared pigment. In both crystal structures, the occupancy position of Fe/Co ions is in the Mg^2+^ site. The Mg^2+^ ions become MgO_6_ via bonding with six O^2−^ atoms. Each TeO_6_ octahedron shares edges with (Mg/Fe/Co)O_6_ octahedra. However, four edges of the (Mg/Fe/Co)O_6_ octahedron share with the same neighboring octahedra, and the TeO_6_ octahedra are connected with another two edges. Moreover, the TeO_6_ octahedra appear to be regular with average Te─O_6_ bond lengths of 1.952 Å, but the (Mg/Fe/Co)O_6_ octahedra were distorted with M─O bond lengths in the range 1.996–2.275 Å.

**FIGURE 3:**
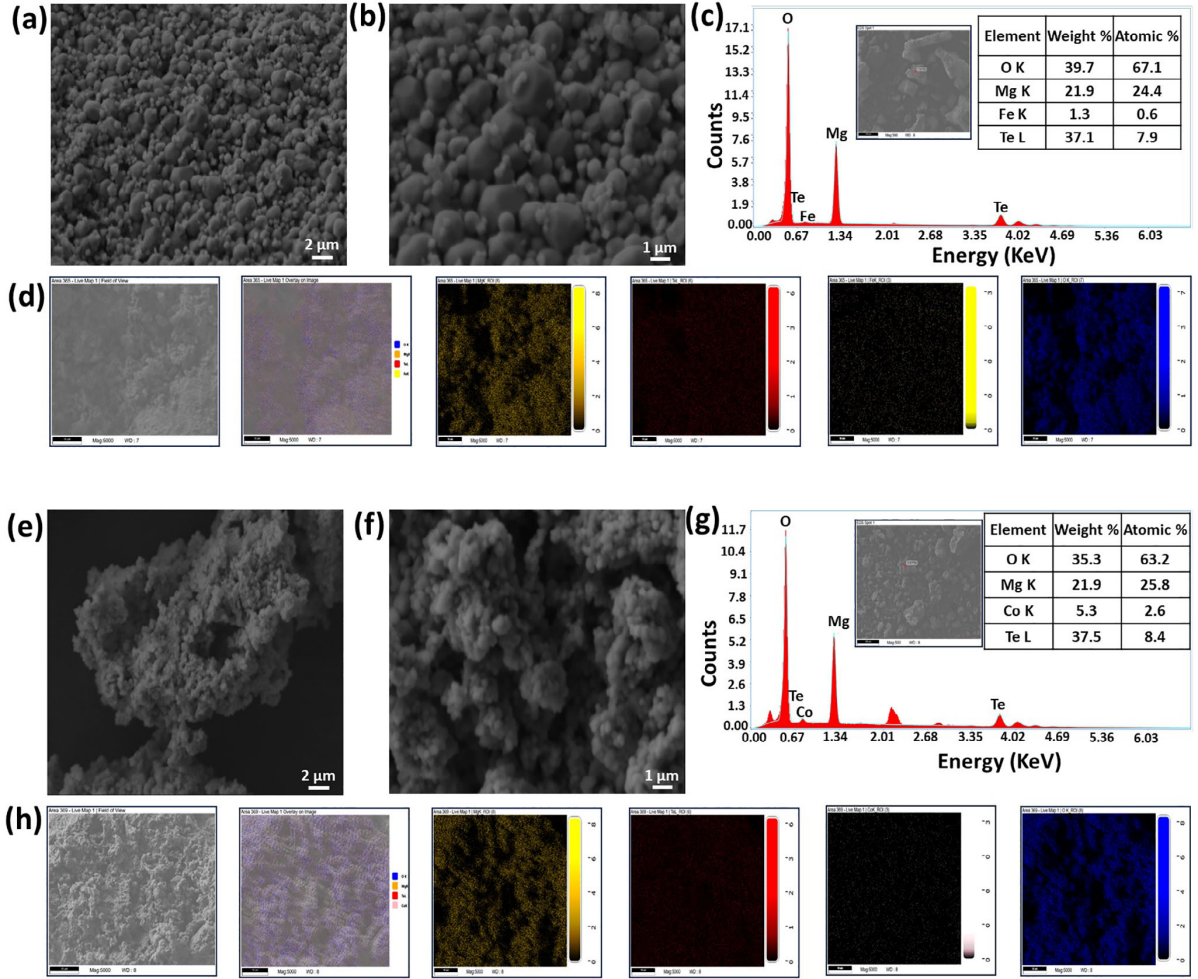
(a,b) SEM images of Mg_2.85_Fe_0.15_TeO_6_, (c) EDX spectrum of Mg_2.85_Fe_0.15_TeO_6_ pigment, (d) Elemental mapping of the Mg_2.85_Fe_0.15_TeO_6_ pigment, (e,f) SEM images of Mg_2.85_Co_0.15_TeO_6_, (g) EDX spectrum of Mg_2.85_Co_0.15_TeO_6_ pigment, (h) Elemental mapping of the Mg_2.85_Co_0.15_TeO_6_ pigment. [Inset tables in Figure 3c,g corresponding to the elements and their compositions].

**FIGURE 4:**
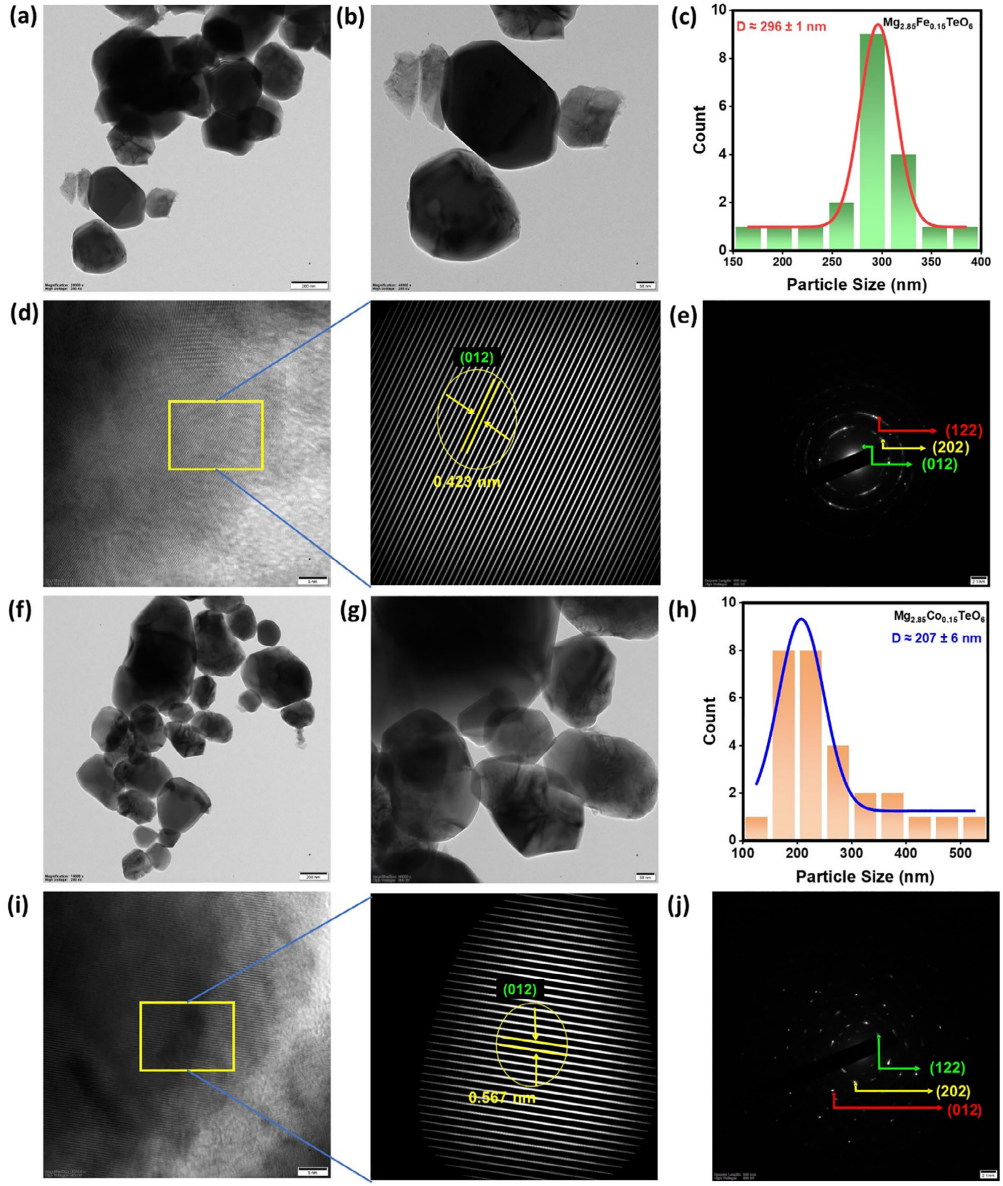
(a,b,f,g) TEM images, (c,h) Histograms indicating the particle size distribution, (d,i) HRTEM and corresponding enlarged portions of HRTEM, and (e,j) SAED patterns of Mg_2.85_Fe_0.15_TeO_6_ and Mg_2.85_Co_0.15_TeO_6_ pigments, respectively.

The morphology and elemental composition of the prepared pigments were analyzed. Scanning electron microscope (SEM) images of the prepared Mg_2.85_Fe_0.15_TeO_6_ (Figure 3a,b) and Mg_2.85_Co_0.15_TeO_6_ (Figure 3e,f) pigments revealed a large number of spherical particles agglomerated together with voids. The morphologies of the produced nano pigments were obvious in the samples obtained through the SC route [20]. Importantly, the observed air voids demonstrated outstanding reflectance capabilities through effective multiple scattering of sunlight [21, 22]. In SC synthesis, the formation of voids and pores is primarily driven by the rapid evolution of gaseous products during the highly exothermic redox reaction. An intense release of gases (such as NO_x_, CO_2_, and H_2_O) during the combustion of the fuel and metal nitrate is the primary driver of void formation. This rapid outgassing acts like a “blowing agent,” creating a voluminous and highly porous structure in the materials [23]. The energy dispersive X‐ray (EDX) spectrum for both pigments (Figure 3c,g) verifies the constituents of the samples, containing Mg, Te, Fe, Co, and O, with atomic ratios, suggesting negligible elemental loss or contamination during the synthesis process. A peak at 2.1 keV (Lα_1_) (Figure 3g) was the most common gold peak, arising from a gold sputter coating. Additionally, the EDX mappings for both pigments show a homogeneous distribution of these different atoms throughout the sample, highlighting the successful incorporation of doped TM ions into the Mg_3_TeO_6_ lattice (Figure 3d,h). Transmission electron microscopic (TEM) images of the Mg_2.85_Fe_0.15_TeO_6_ and Mg_2.85_Co_0.15_TeO_6_ pigments are shown in Figure 4a,b,f,g. As witnessed from the images, particles are almost spherical in shape and are consistent with corresponding SEM results. In addition, the average particle size (*D*) of the prepared Mg_2.85_Fe_0.15_TeO_6_ and Mg_2.85_Co_0.15_TeO_6_ pigments (Figure 4c,h) was estimated and found to be 296 ± 1 and 207 ± 6 nm, respectively. The obtained *D* values (> 100 nm) have higher NIR scattering/reflection efficiency due to Mie resonances [24]. The noticed maximum reflectance of the pigments stems from their particle size and the shape of the materials. In addition, high‐resolution transmission electron microscopic (HRTEM) images and their enlarged portions of Mg_2.85_Fe_0.15_TeO_6_ and Mg_2.85_Co_0.15_TeO_6_ pigments (Figure 4d,i) showcase clear lattice fringes, which are indexed to the most prominent (012) plane of hexagonal Mg_3_TeO_6_ with an interplanar spacing of ~ 0.423 and 0.567 nm, respectively. The selected area electron diffraction (SAED) patterns (Figure 4e,j) of the best‐performing pigments exhibit several orders of diffraction spots in the form of rings, which represent the high crystallinity of the pigments, which are indexed to (012), (202), and (122) planes.

**FIGURE 5:**
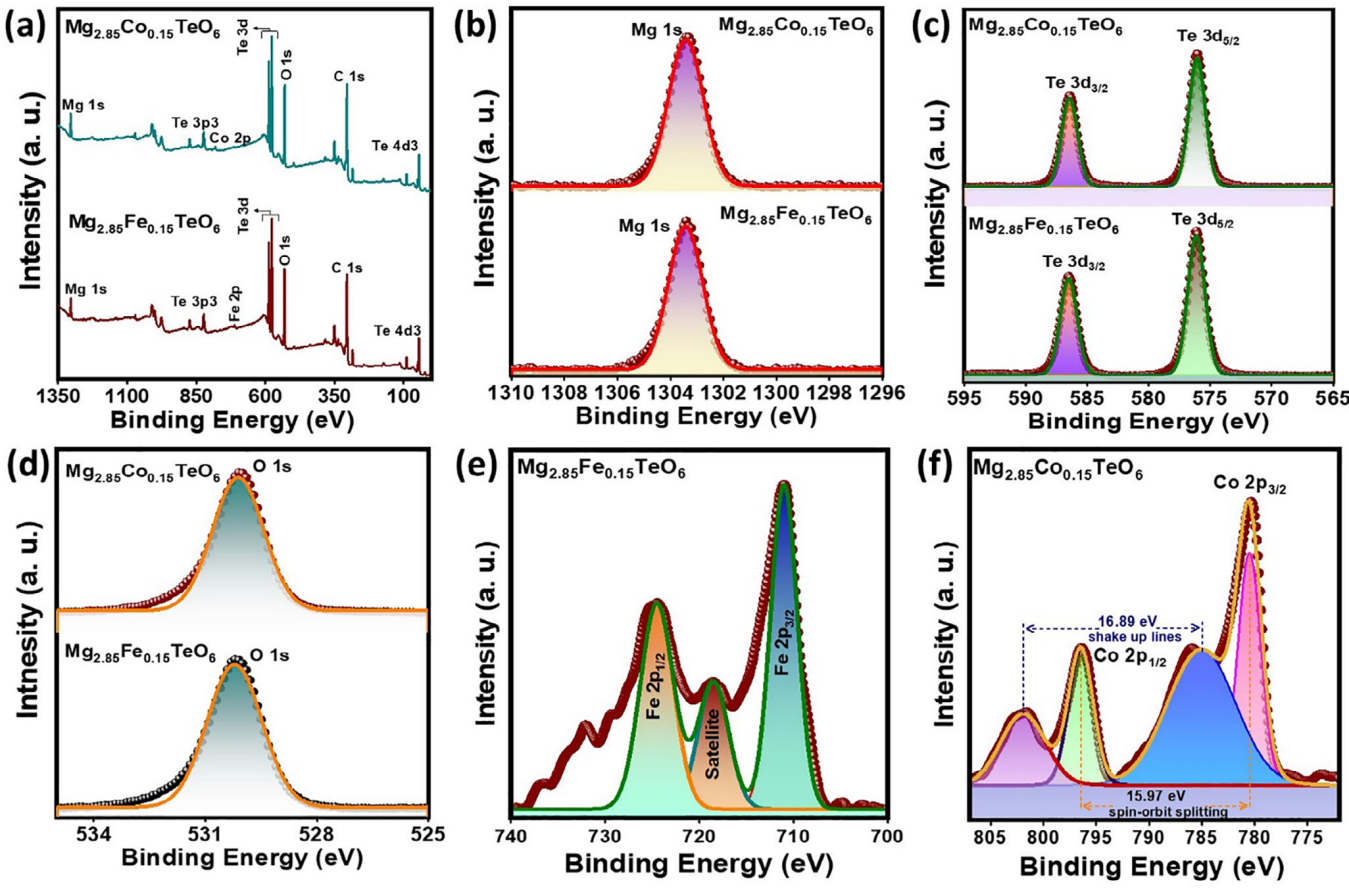
(a) XPS scan survey of the prepared Mg_2.85_Fe_0.15_TeO_6_ and Mg_2.85_Co_0.15_TeO_6_ pigments, High‐resolution XPS spectra of (b) Mg 1s, (c) Te 3d, (d) O 1s, (e) Fe 2p, and (f) Co 2p.

The functional groups or chemical bonds associated with the prepared pigments were analyzed. Fourier transform infrared (FTIR) spectra of the prepared pristine, Mg_2.85_Fe_0.15_TeO_6_, and Mg_2.85_Co_0.15_TeO_6_ pigments are depicted in Figure S3. As evident from the figure, the FTIR spectra of the prepared pigments revealed a peak at around 450 cm^−1^ associated with the stretching vibration of O–Mg/Fe/Co–O in the octahedral site. All the pigments revealed a C–H stretching‐related peak at 560 cm^−1^. Moreover, a peak at 710 cm^−1^ was attributed to O–M–O in the band. No obvious shift or any additional bands in the IR absorption bands were detected after doping TM ions, signifying that TM ions will not induce any structural variations and that all of the prepared pigments were pure phase. X‐ray photoelectron spectroscopy (XPS) survey scan spectra of prepared Mg_2.85_Fe_0.15_TeO_6_ and Mg_2.85_Co_0.15_TeO_6_ pigments were displayed in Figure 5a. The spectra revealed the existence of Mg, Te, Fe, and Co elements. The deconvoluted high‐resolution spectra attest to a single peak of Mg 1s in both Mg_2.85_Fe_0.15_TeO_6_ and Mg_2.85_Co_0.15_TeO_6_ pigments at a binding energies of 1303.42 and 1303.41 eV (Figure 5b), respectively, which implies that Mg has a +2‐oxidation state in the prepared pigments [25]. As seen from the XPS spectra of Te 3d (Figure 5c) in the Mg_2.85_Fe_0.15_TeO_6_ pigment, the two binding energy peaks were witnessed at 586.52 and 576.13 eV; however, Mg_2.85_Co_0.15_TeO_6_ pigment also showcases two peaks at 586.45 and 576.07 eV, which are linked with Te 3d_3/2_ and Te 3d_5/2_, respectively, in both the pigments. The obtained results specify the + 6‐oxidation state of Te [26]. The O 1s peak of Mg_2.85_Fe_0.15_TeO_6_ and Mg_2.85_Co_0.15_TeO_6_ pigments, shown in Figure 5d, revealed a peak at 530.21 and 530.12 eV, respectively. The obtained O 1s peak mainly corresponds to lattice oxygen in the hexagonal structure. High‐resolution Fe 2p XPS spectra (Figure 5e) exhibit peaks at 724.52 and 711.05 eV, which are related to Fe 2p_1/2_ and Fe 2p_3/2_, respectively. In addition, a satellite peak was found at binding energy of 718.55 eV, representing Fe 2p_3/2_ [27]. The obtained result confirms the + 2‐oxidation state of Fe. However, two main peaks appeared related to Co 2p in the Mg_2.85_Co_0.15_TeO_6_ pigment (Figure 5f) at 780.48 and 796.45 eV, which were attributed to Co 2p_3/2_ and Co 2p_1/2_, respectively. The elucidated result confirms the +2‐oxidation state of the Co. The energy difference between the Co 2p_3/2_ and Co 2p_1/2_ levels has been measured to be 15.97 eV, which aligns with the divalent state of cobalt uniformly surrounded by the oxygen octahedron [28]. The results presented above indicate that Co atoms have effectively integrated into the octahedral positions of the Mg_3_TeO_6_ matrix as a substitutional dopant, without the emergence of any identifiable impurity phase or Co clusters. In addition, satellite peaks at binding energies of 785.03 eV (2p_3/2_) and 801.92 eV (2p_1/2_) with a difference of 16.89 eV are witnessed (Figure 5f). The noticed satellite peaks were mainly ascribed to crystal field splitting for Co^2+^ in an octahedral crystal field environment [26].

**FIGURE 6:**
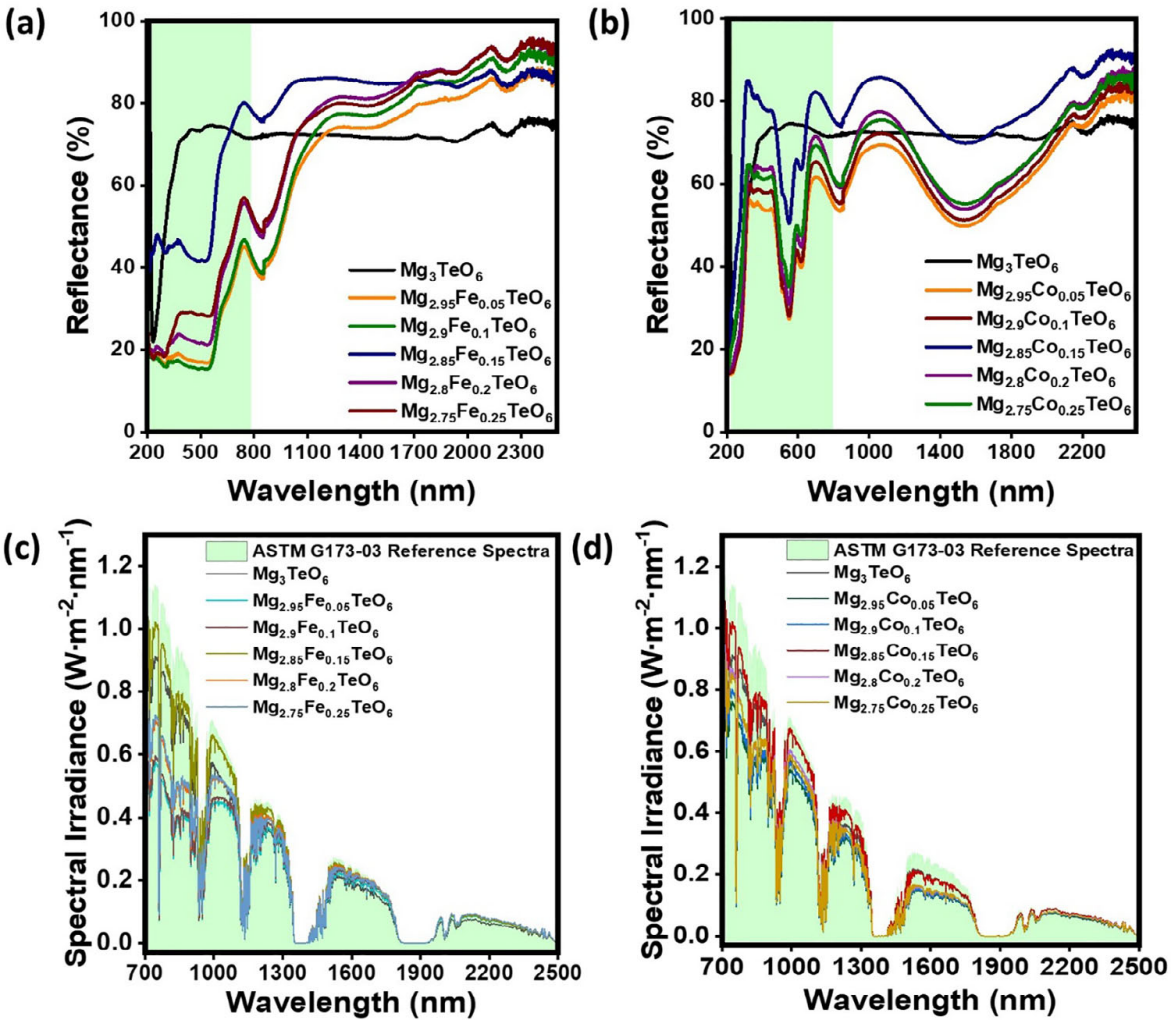
UV–vis–NIR reflectance and solar reflectance spectra of the prepared (a,c) Mg_3−_ *_x_* Fe*_x_* TeO_6_ (*x* = 0.05–0.25 wt.%) and (b,d) Mg_3−_ *_x_* Co*_x_* TeO_6_ (*x* = 0.05–0.25 wt.%) pigments, respectively.

The ultraviolet (UV)‐visible spectra of pristine and Mg_3−_ *_x_* Fe*_x_* TeO_6_ (*x* = 0.05–0.25 wt.%) pigments in the region 200–800 nm were depicted in Figure 6a. It can be noticed that the Mg_3_TeO_6_ material displayed minimal light absorbance in the UV–vis region (i.e., the majority of the light is reflected), which indicates the white appearance of the Mg_3_TeO_6_ sample. However, the reflectance spectra of Mg_3−_ *_x_* Fe*_x_* TeO_6_ (*x* = 0.05–0.25 wt.%) pigments in the same region display (Figure 5a) several absorption peaks at 392, 441, 520, and 630 nm, which are ascribed to ^6^A_1_ (^6^S)→^4^E (^4^D), ^6^A_1_ (^6^S)→^4^T_2_ (^4^D), ^6^A_1_ (^6^S)→^4^T_2_ (^4^G), and ^6^A_1_ (^6^S)→^4^T_1_ (^4^G) transitions of chromophore Fe^2+^ ions in the Mg_3_TeO_6_ lattice structure, respectively. The observed absorption peaks indicate that the incorporated Fe^2+^ chromophore ions into octahedra sites of the Mg_3_TeO_6_ lattice play a significant role in the material's ability to absorb certain portions of UV–vis light. This phenomenon contributes to the development of distinct hues in the prepared pigments, highlighting the potential for enhancing color characteristics through careful engineering of the crystal structure via doping TM ions. Similarly, diffuse reflectance spectra of Mg_3−_ *_x_* Co*_x_* TeO_6_ (*x* = 0.05–0.25 wt.%) pigments (Figure 6b) show remarkable absorption peaks in the visible region at 565, 610, and 653 nm, which are ascribed to ^4^A_2_ (F) → ^2^A_1_(G), ^4^A_2_(F) → ^4^T_1_(P) and ^4^A_2_(F) → ^2^E(G) ligand field transitions, respectively [29], which involve a crystal‐field split in the 3d levels of Co^2+^ substituting Mg^2+^ site of Mg_3_TeO_6_. The color of Mg_3−_ *_x_* Co*_x_* TeO_6_ pigments is attributed to typical *d*–*d* transitions in high‐spin states of Co^2+^ in the octahedral positions of the Mg^2+^ in the Mg_3_TeO_6_ matrix.

The NIR reflectance property of the prepared pristine and Mg_3‐x_Fe_x_TeO_6_ (*x* = 0.05–0.25 wt.%) pigments in a range of 800–2500 nm was analyzed. As seen from Figure 6a and Figure S4a, the pristine Mg_3_TeO_6_ material exhibits an average NIR reflectance of ∼81%. However, the enhancement in the NIR reflectance was witnessed when the chromophore Fe^2+^ ions were substituted in the octahedral Mg^2+^ site in the lattice. Moreover, among the Fe‐doped pigments, the Mg_2.85_Fe_0.15_TeO_6_ pigment established the average reflectance in the NIR region (800–2500 nm) of ∼ 86% (i.e., ∼16.4% higher than a pristine sample). The chromophore Fe^2+^ doping not only enhanced the NIR reflectance but also enriched the hue of the pigment. As the concentration of chromophore Fe^2+^ ions in the Mg_3_TeO_6_ matrix increased beyond 0.15 wt.%, the NIR reflectance was decreased slightly; the synthesized pigments still retain a relatively higher NIR reflectance compared with previously reported literature. For instance, Robert Ianoș et al. (2024) synthesized NIR‐reflective Ce_1−x_Pr_x_O_2_ red‐brown pigments by solid‐state reactions. The total solar reflectance of ceramic Ce_1‐x_Pr_x_O_2_ pigment was also achieved to be 86% [30]. Nonetheless, the pigment that has been prepared incorporates precious metals, resulting in high costs for its use [14]. It is worth noting that the prepared Mg_3−_ *_x_* Fe*_x_* TeO_6_ (*x* = 0.15 wt.%) pigment attains the same reflectance with a brilliant brown color at a low cost and sidelines the other similar pigments. In addition, Jiang et al. successfully synthesized red‐shaded CaFe_2‐x_In_x_O_4_ (0 ≤ x ≤ 1) pigment via effective substitution of In^3+^ into FeO_6_ octahedra in the host matrix. The prepared pigments exhibit an overall NIR reflectance of ∼ 87% [31]. However, Gopalan et al. [32] partially replaced the octahedral geometry of Fe^3+^ chromophore by Al^3+^ in the pigment composition (ZnFe_1.9_Al_0.1_O_4_), which resulted in NIR reflectance of 58%. From the above analysis, it was clear that the octahedral site‐manipulated Mg_2.85_Fe_0.15_TeO_6_ pigment demonstrated the accomplished improvement in the NIR‐reflectance as compared to its counterparts. Figure 6b and Figure S4b show the NIR reflectance property of the prepared Mg_3−_ *_x_* Co*_x_* TeO_6_ (*x* = 0.05–0.25 wt.%) pigments. As seen from the figure, the average reflectance in the NIR region (800–2500 nm) of 86% in the Mg_2.85_Co_0.15_TeO_6_ pigment was witnessed. As compared with pristine material, the optimized pigment showed an enhancement in the NIR reflectance of about 6.6%. The reflectance intensity was diminished when the Co^2+^ concentration was increased beyond 0.15 wt.%, but retained the hue of the material. Similarly, Yun et al. [33] studied the blue pigment based on Mg_1–_ *_x_* Co*_x_* TiO_3_, where the octahedral site was engineered via Co‐doping. The synthesized pigment shows a high NIR solar reflectance of 78.9%. The NIR reflectance of various Fe‐ and Co‐doped pigments reported in previous literature was tabulated in Table S3. As witnessed, the best‐performing pigments effectively reflect the maximum part of the solar radiation as compared to previous reports, which is attributed to the strategic doping of chromophores in an octahedral site of corundum‐structured Mg_3_TeO_6_ pigment. The solar reflectance spectra of the pristine and Mg_3−_ *_x_* A*_x_* TeO_6_ (A = Fe and Co; *x* = 0.05–0.25 wt.%) pigments were also studied and depicted in Figure 6c,d. A significant amount of solar irradiance spectral concealment was witnessed in both series of pigments. The obtained results demonstrated that the prepared pigments can be used as a cool coating material for energy‐saving applications to combat UHIs.

**FIGURE 7:**
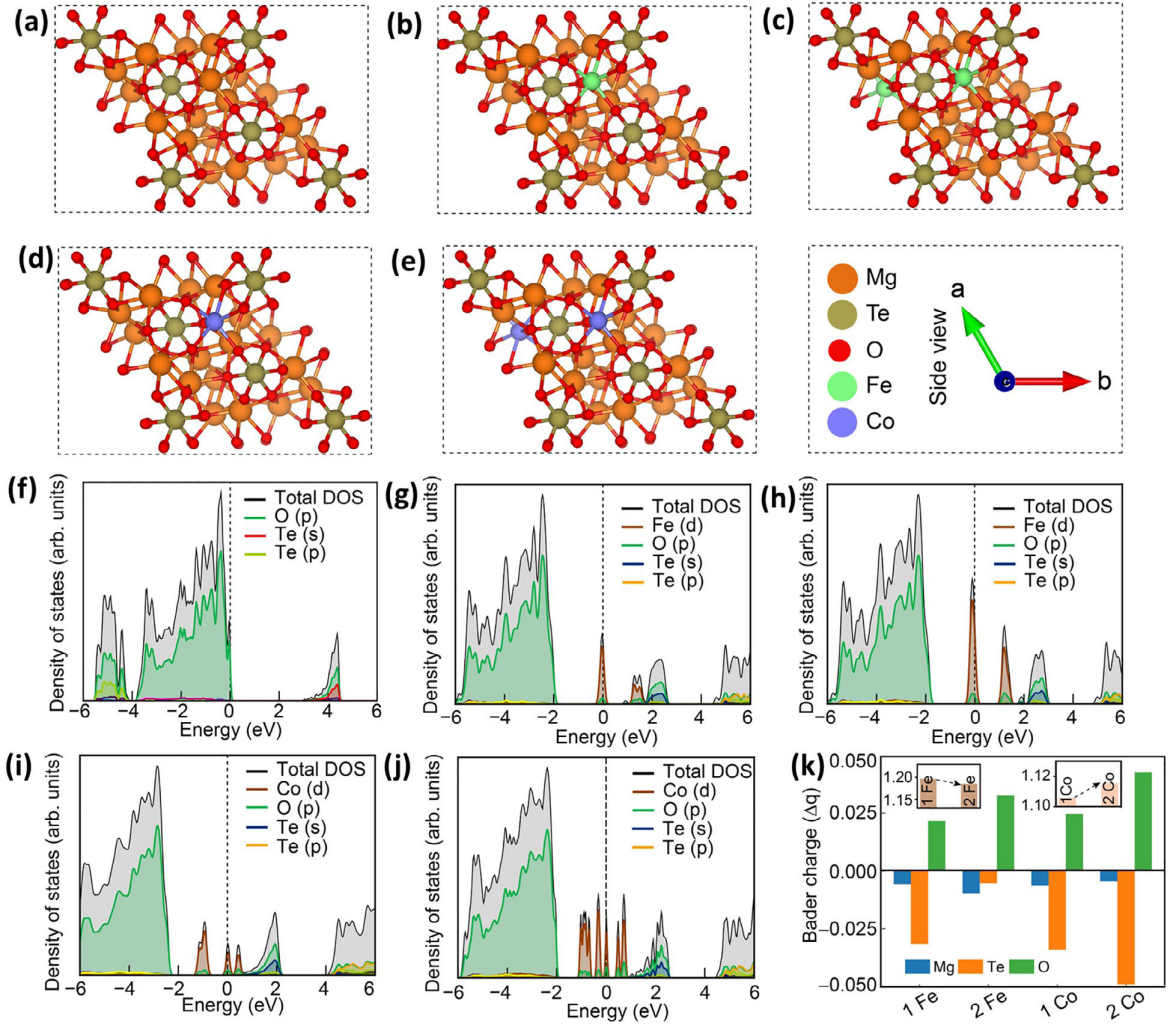
DFT‐optimized crystal structure of (a) undoped Mg_3_TeO_6_, (b) Mg_3_TeO_6_ with low Fe concentration, (c) Mg_3_TeO_6_ with high Fe concentration, (d) Mg_3_TeO_6_ with low Co concentration, and (e) Mg_3_TeO_6_ with high Co concentration, the DOS of (f) undoped Mg_3_TeO_6_, (g) Mg_3_TeO_6_ with low Fe concentration, (h) Mg_3_TeO_6_ with high Fe concentration, (i) Mg_3_TeO_6_ with low Co concentration, and (j) Mg_3_TeO_6_ with high Co concentration, (k) The change in charge transfer in doped systems with respect to the undoped system, calculated from the Bader charge analysis. The insets show the charge transfer toward Fe with increasing Fe concentration and the charge transfer from Co with increasing Co concentration.

The significant increase in NIR reflectance observed experimentally upon Fe and Co‐doping in the Mg_3_TeO_6_ pigments was well supported by the density functional theory (DFT) results. The side view of DFT optimized Mg_3_TeO_6_ structure is shown in Figure 7a. The optimized lattice parameters are obtained to be *a* = *b* = 8.677 Å and *c* = 10.443 Å. The optimized lattice parameters of Fe‐doped Mg_3_TeO_6_ were found to be *a* = 8.679 Å, *b* = 8.675 Å, and *c* = 10.439 Å, as shown in Figure 7b. For higher Fe concentration, the optimized lattice parameters are *a* = 8.592 Å, *b* = 8.588 Å, and *c* = 10.359 Å, as shown in Figure 7c. The optimized lattice parameters of Co‐doped Mg_3_TeO_6_ are *a* = 8.693 Å, *b* = 8.648 Å, and *c* = 10.470 Å, as shown in Figure 7d. The optimized lattice parameters of higher Co doping concentration are *a* = 8.743 Å, *b* = 8.768 Å, and *c* = 10.484 Å, as shown in Figure 7e. It was clear that the theoretical lattice parameters were well aligned with the experimentally derived crystal structure parameters estimated from the Rietveld refinements.

The DOS plots (Figure 7f–j) revealed how doping Mg_3_TeO_6_ with Fe and Co affects its electronic properties. In the undoped Mg_3_TeO_6_, the DOS is primarily contributed by O(p) and Te(s) orbitals near the Fermi level. However, O(p) orbitals cross the Fermi level in a minimal amount, as shown in Figure 7f. Upon introducing Fe at low concentration, Fe(d) orbitals emerge exactly at the Fermi level with a small contribution coming from O(p) orbitals. Additionally, hybridization of O(p) and Te(s) orbitals and O(p) and Fe(d) orbitals is observed within 0 to 4 eV, as shown in Figure 7g. The hybridization between O(p) and Fe(d)/Co(d) states facilitates selective absorption in the visible range while allowing stronger reflection in the NIR region. At higher Fe concentration, as shown in Figure 7h, these Fe(d)‐states become more pronounced, suggesting stronger electronic interactions and increased charge accumulation on Fe. Co‐doping shows a similar trend. Co(d) orbitals mainly contribute with a small contribution at the Fermi level, and hybridization of O(p) and Te(s) orbitals and O(p) and Co(d) orbitals is observed within 0 to 4 eV, as shown in Figure 7i. Increasing Co concentration increases the contribution of Co d‐states at the Fermi level, as shown in Figure 7j. However, this contribution is slightly less compared to the higher Fe concentration case, indicating a lower charge transfer to Co compared to Fe.

**FIGURE 8:**
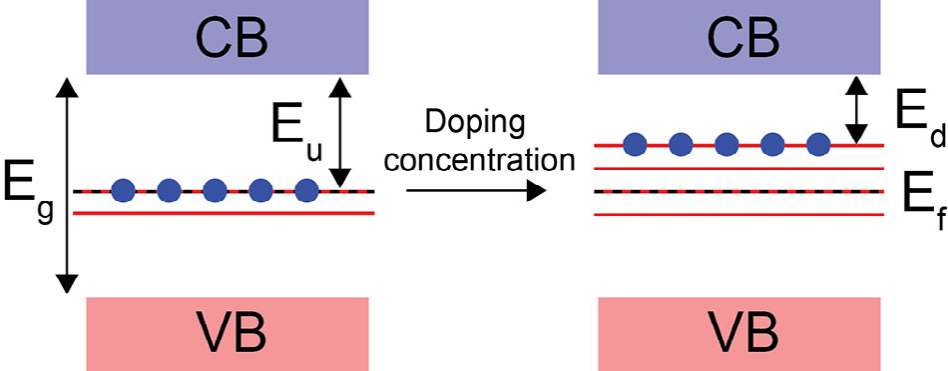
Schematic illustration of mid‐gap states within the bandgap for both the undoped, low‐doping‐concentration, and the higher‐doping‐concentration cases. Mid‐gap states are represented by solid red lines, while the Fermi level is indicated by a black dotted line. In the undoped or low‐doping case, the energy separation between the Fermi level and the conduction band minimum (E_u_) is higher than the corresponding separation (E_d_) observed at higher doping levels. As a result, lower‑energy NIR excitation is sufficient to promote electrons from the mid‐gap states into the conduction band with increasing doping concentration. The enhanced accessibility of electronic transitions leads to increased NIR reflectance in the higher‐doping case.

In the undoped material, a small number of mid‐gap states already exist inside the bandgap, but they are too few to significantly affect NIR reflectance. As a result, NIR photons have a limited ability to excite electrons, and the reflectance remains weak. When dopants are introduced, the number of mid‐gap states increases, and their presence changes the electronic structure. Electrons can now move more easily from the valence band into these mid‐gap states, and from the mid‐gap states into the conduction band. These additional pathways match the energy of NIR photons, allowing more sub‑bandgap transitions and leading to stronger NIR reflectance. The higher the dopant concentration, the greater the density of mid‐gap states, and the stronger the charge transfer, both of which directly enhance NIR reflectance compared to the undoped case. The whole mechanism has been schematically represented in Figure 8.

To further confirm this, we perform the Bader charge analysis for both the undoped and doped cases (Figure 7k). In the undoped system, O atoms carry a higher negative charge. Upon Fe doping, the negative charge on O atoms becomes lower, indicating oxidation. At the same time, the positive charges on Mg and Te atoms decrease, indicating reduction. This reflects a charge transfer from Mg and Te to O atoms. A similar pattern is also observed upon Co doping. With increased Fe concentration (indicated by 2Fe), the positive charges on Mg and Fe decrease, while Te shows an increase in positive charge. The negative charge on oxygen also decreases. This suggests that electrons were transferred from Te and O to Fe and Mg atoms. On the other hand, when Co concentration increases (indicated by 2Co), Mg and Te continue to show lower positive charges. Cobalt shows a higher positive charge, and O becomes more oxidized. This indicates electron transfer from oxygen and cobalt to magnesium and tellurium. Both Fe and Co doping result in electron transfer from O to Mg and Te. Increasing Co concentration strengthens this effect, with Co transferring charge to Te. Increasing Fe concentration leads to greater electron transfer toward Fe from Te. In comparison, Fe shows a higher charge transfer than Co. This correlates with the experimentally observed higher NIR reflectance in Fe‐doped MgTeO_6_ compared to Co‐doped ones.

**FIGURE 9:**
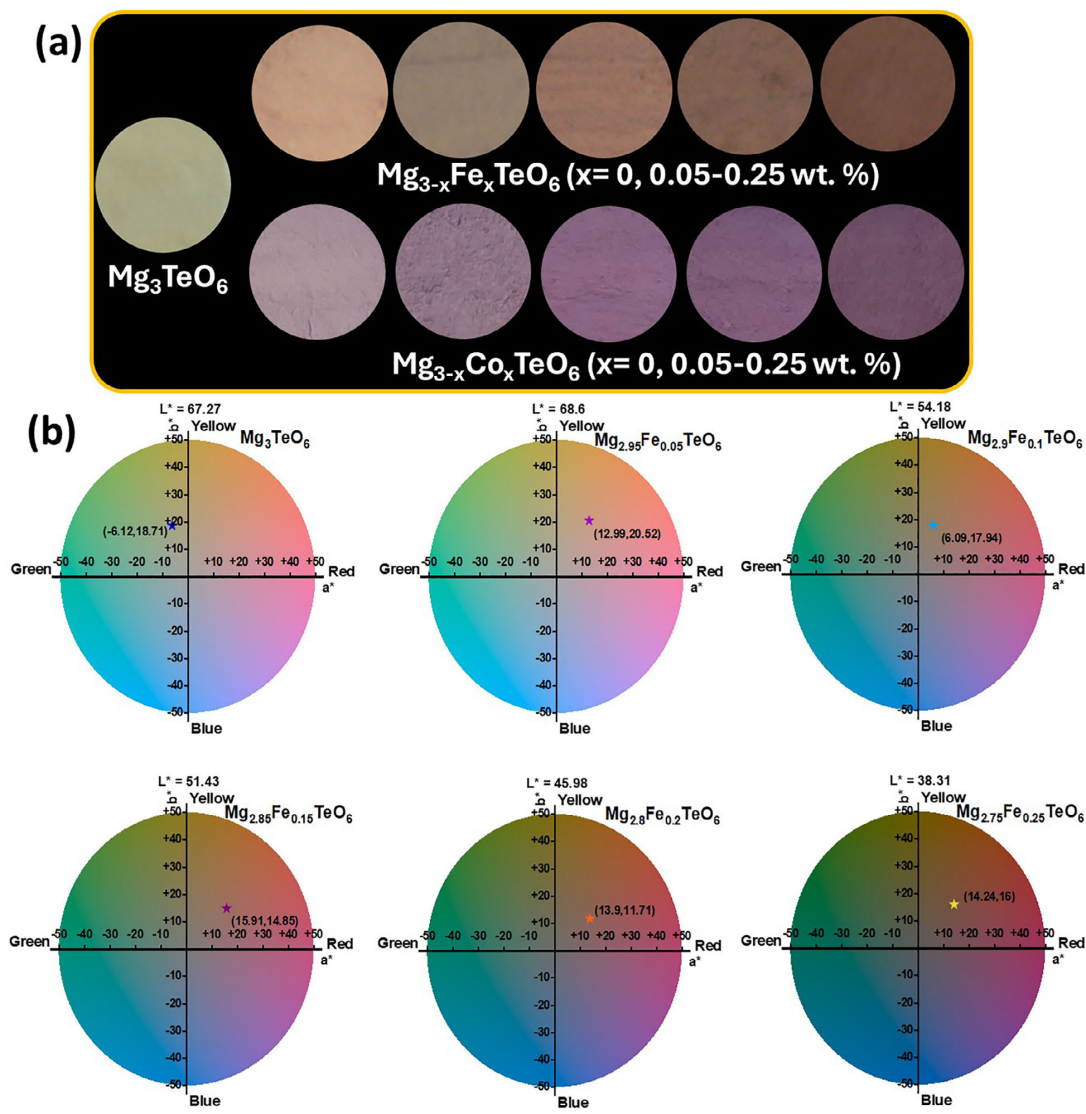
(a) Photographic images of the prepared pristine, Mg_3−_ *_x_* A*_x_* TeO_6_ (A = Fe and Co; *x* = 0.05–0.25 wt.%) pigments and (b) CIE 1976 L^*^a^*^b^*^ diagrams of the prepared pristine and Mg_3−_ *_x_* Fe*_x_* TeO_6_ (x = 0.05–0.25 wt.%) pigments.

The photographic images of the prepared pristine and Mg_3−_ *_x_* A*_x_* TeO_6_ (A = Fe and Co; *x* = 0.05‐0.25 wt.%) pigments are depicted in Figure 9a. The integration of the Fe and Co chromophores into the octahedral positions of the Mg^2+^ site in the Mg_3_TeO_6_ matrix can evidently alter the hue of the pigments. The noticeable alteration in color was primarily attributed to the asymmetrical coordination environments created around the TM ions in the octahedral site, leading to modifications in the selection rules and enabling the detection of forbidden electronic transitions, which in turn cause variations in the positions and intensities of the absorption bands in the optical spectra. To validate the above observations, the photometric properties of the prepared pigments were analyzed. Figure 9b depicts the Commission Internationale de l'éclairage (CIE) 1976 L^*^a^*^b^*^ diagrams of the prepared pristine and Mg_3−_ *_x_* Fe*_x_* TeO_6_ (*x* = 0, 0.05–0.25 wt.%) pigments. As witnessed from the figure, the pristine Mg_3_TeO_6_ material exhibits an L^*^ value of 67.27 and (a^*^ and b^*^ values of −6.12 and 18.71, respectively). Nevertheless, Mg_3−_ *_x_* Fe*_x_* TeO_6_ (*x* = 0.05–0.25 wt.%) pigments showcase a decrement in the L^*^ values from 67.27 to 38.31, which demonstrates that the pigments have become darker when chromophore Fe^2+^ ions were substituted in the Mg^2+^ site. Moreover, the best‐performing Mg_2.85_Fe_0.15_TeO_6_ pigment exhibits the highest a^*^ value of 15.91, which indicates that the red‐green degree inclines toward red; however, prepared pigments retain the b^*^ (blue to yellow coordinate) values around 10–25. CIE 1976 L^*^a^*^b^*^ diagrams of the prepared pristine and Mg_3−_ *_x_* Co*_x_* TeO_6_ (*x* = 0, 0.05‐0.25 wt.%) pigments are shown in Figure S5. As evident from the figure, L^*^ values of the Mg_3−_ *_x_* Co*_x_* TeO_6_ (*x* = 0.05–0.25 wt%) pigments continuously decreased (from 67.27 to 39.26), as the concentration of Co^2+^ ions increased, indicating that lightness is diminishing and visually faint. In addition, the a^*^ and b^*^ values of the pigments were completely tuned from the green–yellow region to the red–blue region after successfully incorporating Co^2+^ ions in the Mg_3_TeO_6_ lattice. Furthermore, chroma (C^*^) and hue angle (*H°*) values of the prepared pigments were also estimated and tabulated in Table S4. The human visual inspection of the photographic images of the prepared pigments is well‐matched with the estimated color coordinates.

The chemical stability performance of the best‐performing Mg_2.85_Fe_0.15_TeO_6_ and Mg_2.85_Co_0.15_TeO_6_ pigments was analyzed. Around 1 g of pigment powder was immersed in 50 mL of an aqueous solution containing 5% acids (HNO_3_ and HCl) or alkali solutions (NaOH and NH_4_OH), and distilled water for 30 min. After treatment, the powders were filtered, thoroughly rinsed with distilled water to remove residual reagents, and dried in an oven at 60°C for 3 h. The color integrity of the dried samples was examined using CIE L^*^a^*^b^*^ color coordinates. Table 1 summarizes the CIE L^*^a^*^b^*^ color coordinates of the prepared pigments soaked and vibrated in the water, 5% acids (HNO_3_, and HCl), and alkali solutions (NaOH and NH_4_OH). As witnessed from the table, pigments showcase no significant changes in the color coordinates after chemical treatment. This was further validated by the estimated color difference (ΔE^*^) values of the pigments. The Mg_2.85_Fe_0.15_TeO_6_ pigment showcases a maximum ΔE^*^ value of 3.48 against NH_4_OH treatment when compared with other chemicals. For the Mg_2.85_Co_0.15_TeO_6_ pigment, the maximum ΔE^*^ value (i.e., 3.92) was obtained in the NaOH‐treated pigment. The determined ΔE^*^ values, which were significantly below the allowable threshold (i.e., ΔE^*^ ≤ 5), indicate that the prepared pigments demonstrate chemical stability when exposed to different acids and bases. In addition, the photostability of the best‐performing pigments was also assessed when exposed to UV 365 nm and IR lamp light. The established ΔE^*^ values were found to be around 3.6 and 3.8 upon UV 365 nm and IR lamp illumination, respectively for Mg_2.85_Fe_0.15_TeO_6_ pigment. Similarly, Mg_2.85_Co_0.15_TeO_6_ pigment also demonstrated ΔE^*^ values of 1.43 and 1.06 against UV 365 nm and IR lamp illumination, respectively, which were beyond the standard limit. Besides, the developed pigments have outstanding thermal stability due to the elevated calcination temperatures, which means they can endure high temperatures without changing their color properties. In fact, C^*^ and *H°* values of the best‐performing pigments after chemical and light treatment were also calculated and listed in Table 1. The aforementioned results demonstrated that the prepared pigments exhibit excellent acid/alkali resistance, photostable, as well as thermal stability. Based on these properties, the prepared pigments were envisaged as the best candidates for energy‐saving coatings.

**TABLE 1:** CIE L^*^a^*^b^*^ color coordinates, C^*^ and *H°* values of the best‐performing Mg_2.85_Fe_0.15_TeO_6_ and Mg_2.85_Co_0.15_TeO_6_ pigments against chemical and photo stability tests.

Materials	Treatment	*L*	a^*^	b^*^	C^*^	*H°*	ΔE^*^
Mg_2.85_Fe_0.15_TeO_6_	Without Treatment	51.43	15.91	14.85	21.76	43.03	—
	H_2_O	52.71	14.95	12.56	19.53	40.03	2.79
	HNO_3_	52.82	15.36	12.73	19.95	39.65	2.59
	HCl	51.67	13.68	16.31	21.29	50.01	2.67
	NaOH	51.15	14.83	15.61	21.53	46.47	1.35
	NH_4_OH	53.61	13.53	16.16	21.08	50.06	3.48
	Under UV lamp	54.98	15.78	15.86	22.37	45.14	3.69
	Under NIR lamp	52.95	16.86	18.23	24.83	47.24	3.82
Mg_2.85_Co_0.15_TeO_6_	Without Treatment	45.81	16.99	−9.13	19.29	331.75	—
	H_2_O	48.59	16.81	−9.06	19.10	331.68	2.78
	HNO_3_	45.4	17.01	−9.14	19.31	331.75	0.41
	HCl	43.36	16.95	−8.63	19.02	333.02	2.50
	NaOH	42.14	17.25	−10.51	20.20	328.65	3.92
	NH_4_OH	46.14	16.57	−8.02	18.41	334.17	1.23
	Under UV lamp	44.82	16.49	−10.04	19.31	328.66	1.43
	Under NIR lamp	45.63	16.44	−10.02	19.25	328.64	1.06

The thermal conductivity and effusivity of the best‐performing pigment powder were analyzed. The thermal conductivity of Mg_2.95_Fe_0.15_TeO_6_ and Mg_2.95_Co_0.15_TeO_6_ pigments was recorded and found to be 0.06 and 0.08 W/m. K, respectively. The microstructural voids observed in the pigment particles may partially contribute to the low thermal conductivity by increasing phonon scattering and reducing heat transfer pathways. In addition, the IR emissivity spectra of the synthesized pristine and Mg_3−_ *_x_* A*_x_* TeO_6_ (A = Fe and Co; *x* = 0.05–0.25 wt.%) pigments were depicted in Figures S6 and S7. As seen from the figures, the intense IR emissivity of the best‐performing Mg_2.95_Fe_0.15_TeO_6_ and Mg_2.95_Co_0.15_TeO_6_ pigments was found to be 2.1 and 0.66 at 24.23 and 23.49 µm, respectively. The obtained thermal effusivity of the prepared pigments is relatively high compared to conventional roofing materials – for instance, aluminum with a highly polished surface (0.4–0.06), concrete (0.92–0.97), ceramic (0.90–0.94), asbestos board (0.96), and common paints (0.92–0.96). The obtained results indicate that the prepared pigments exhibit low thermal conductivity and high IR emissivity compared to traditional roofing materials. Furthermore, the cooling application of the best‐performing Mg_2.95_Fe_0.15_TeO_6_ and Mg_2.95_Co_0.15_TeO_6_ pigments was also tested against traditional roof sheet materials. The photographic images of the experimental setup used to study the interior cooling applications of pigments‐coated roofs are shown in Figure S8. The plots of interior temperature measurements of a house model provided with Mg_2.95_Fe_0.15_TeO_6_‐coated sheet roof and commercial roofing sheets against different periods are shown in Figure S9. As witnessed, the Mg_2.95_Fe_0.15_TeO_6_‐coated sheet model demonstrated a linear increment in the interior temperature for up to 35 min and reached a steady state (∼39°C) until 1 h of continuous IR light irradiation. Nevertheless, the interior temperature of the bare and other conventional sheet models also exhibits a similar trend; later increases linearly as time elapses and reaches a maximum temperature of ∼45°C. The outcome demonstrated that the pigment‐coated sheets model experienced an almost 6°C cooler interior atmosphere as compared to conventional roofing sheets. From the plots (Figure S10), it was witnessed that the Mg_2.95_Co_0.15_TeO_6_ pigment‐coated roofing system demonstrates ∼42°C at 60 min, while commercial bare‐roofing sheet systems exhibit an interior temperature of ∼45°C. The results indicated that the pigment‐coated roofing system can cool the interior temperature of the house model by around 3°C. The temperature difference between pigment‐coated sheets with commercially available blue, red, and off‐white sheets was found to be ∼ −2, −1.5, and −1°C, respectively. The aforementioned results support the use of the developed pigments, which exhibit outstanding NIR reflectance, for applications in radiative cooling.

**FIGURE 10:**
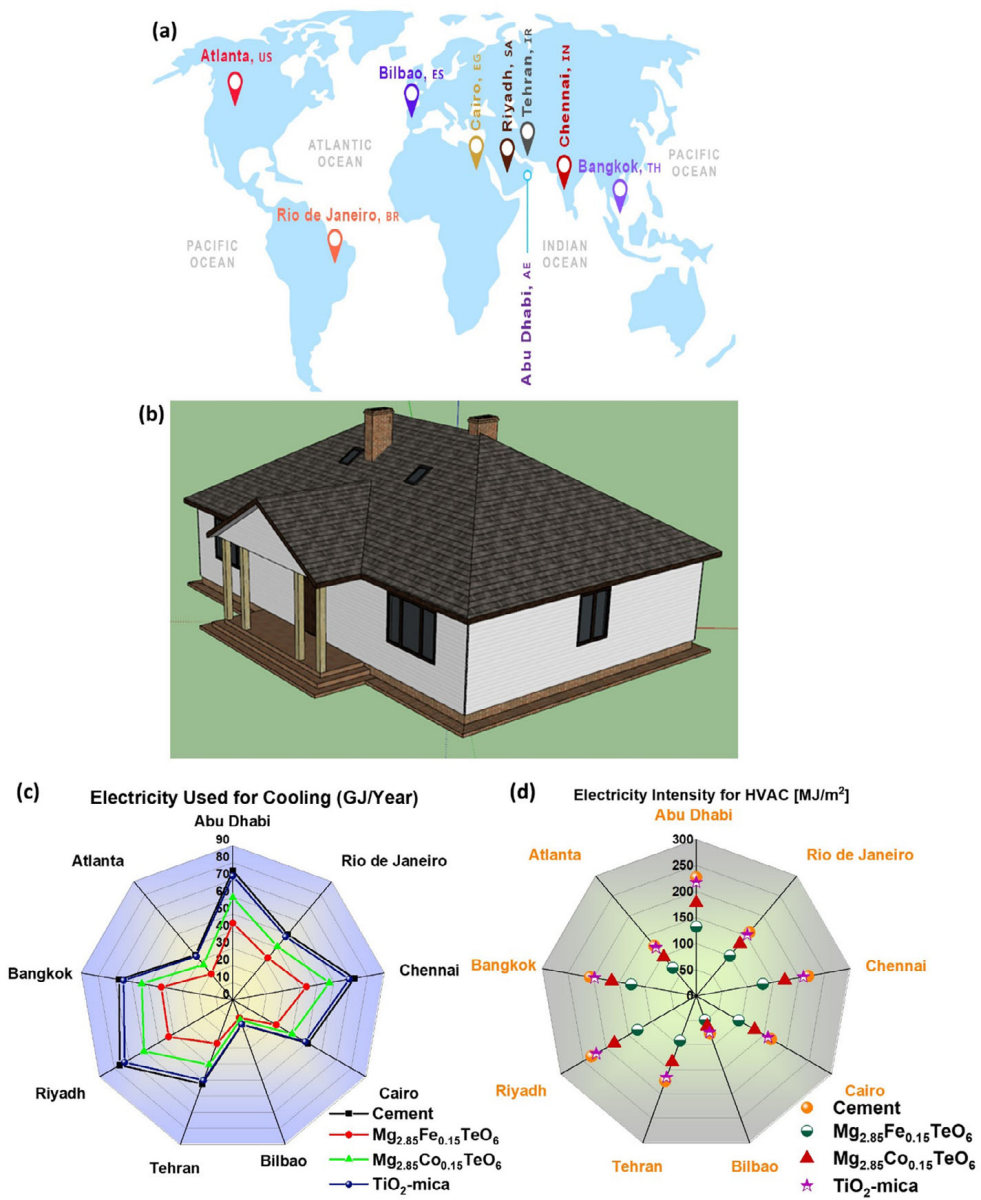
(a) Selected geographical locations for energy consumption simulation, (b) House model designed using an OpenStudio software, (c, and d) The energy consumption demand for cooling and HVAC in the various climate zones, respectively.

The NIR reflectance characteristics and various climate regions greatly influence the energy‐saving effectiveness of cool coatings in real‐world applications [34]. In this view, the influence of cool coatings possessing different NIR reflectance and climate zones on energy‐saving efficiency, i.e., the energy consumption simulations for cool coatings applied in the envelopes of buildings, was studied. The nine cities, including Abu Dhabi (United Arab Emirates), Rio de Janeiro (Brazil), Chennai (India), Cairo (Egypt), Bilbao (Spain), Tehran (Iran), Riyadh (Saudi Arabia), Bangkok (Thailand), and Atlanta (America), with different climate zones (their geographic locations were given in Figure 10a), were selected for energy consumption simulation to quantify the applicability of the prepared pigments. The detailed climate information of these cities was tabulated in Table S5. The OpenStudio software was utilized to design a mid‐size house model with a total area of 400 m^2^ and an indoor occupancy density of 0.125 people/m^2^. Figure 10b shows the designed house model, which adopts a sloped roof and is provided with windows and doors. In addition, the model house is provided with a heating, ventilation, and air conditioning (HVAC) system for cooling/heating by setting the heating and cooling temperatures to 18°C and 26°C, respectively. and it starts cooling/heating when the temperature is higher/lower than 26°C/18°C, respectively. The effective energy‐saving potential of prepared pigments in the specified house model was assessed through *EnergyPlus* software (developed by the U.S. Department of Energy (DOE)) by simulating energy use while preventing solar energy conduction within the model. Figure S11 depicts the total energy demand per conditioned building area of cement, TiO_2_‐mica pearlescent pigment, Mg_2.95_Fe_0.15_TeO_6_, and Mg_2.95_Co_0.15_TeO_6_ pigments coating simulated in the various regions with different climate zones. For instance, Riyadh experiences very hot and dry climatic conditions with high solar insolation (receives a significant amount of direct and intense sunlight). Despite these harsh conditions, the Mg_2.95_Fe_0.15_TeO_6_ and Mg_2.95_Co_0.15_TeO_6_ pigments‐coating house models require maximum energy inputs of 337 and 395 MJ/m^2^, respectively. Notably, the estimated energy demand for these pigments is lower than the energy required for cement (451 MJ/m^2^), as well as for the TiO_2_‐mica pearlescent pigment (440 MJ/m^2^). The lower energy demand in the prepared pigments‐coated models was mainly ascribed to their high NIR‐reflectance as compared to cement and TiO_2_‐mica pearlescent pigment. The envisaged results clearly demonstrated that the prepared pigment coating with high NIR reflectance possesses the potential to save energy. The cooling and HVAC energy consumption demands that respond to assess the climate zones were simulated and shown in Figure 10c,d, respectively. The electricity for cooling in the conventional cement and TiO_2_‐mica pearlescent pigment coating house demands a maximum of 76 and 73 MJ/m^2^ in Riyadh, respectively; whereas 43 and 60 MJ/m^2^ of electricity were required for the cooling system in NIR‐reflectance Mg_2.95_Fe_0.15_TeO_6_ and Mg_2.95_Co_0.15_TeO_6_ pigment coatings. The obtained result indicated that the pigment coatings showcase less electricity demand as compared to conventional cement and TiO_2_‐mica pearlescent pigment coatings. Similarly, the HVAC system (total heating and cooling) also revealed a similar trend, in which cement and TiO_2_‐mica pearlescent pigment coatings required electricity demand in Riyadh is around 232 and 222 MJ/m^2^, respectively; pigment coating, instead, requires less electricity for the HVAC system. These results endorsed that the prepared NIR reflectance pigments for cool coatings possess a favorable energy‐saving ability in mitigating the UHI effect.

## 3 Conclusions

In summary, corundum‐structured Mg_3−_ *_x_* A*_x_* TeO_6_ (A = Fe and Co; *x* = 0.05–0.25 wt.%) pigments with different hues were successfully synthesized. The refined XRD data analysis confirms the formation of hexagonal structure‐type pigments with a centrosymmetric space group $\bar{R_{3}}$. Morphological results revealed a large number of spherical particles agglomerated together with voids. The best‐performing Mg_2.95_Fe_0.15_TeO_6_ and Mg_2.95_Co_0.15_TeO_6_ pigments revealed the average reflectance of ∼86% in the NIR region (800–2500 nm). The Bader charge analysis envisaged that enhanced reflectance in the prepared Mg_2.95_Fe_0.15_TeO_6_ pigment was mainly due to significant transfer of electrons from Te and O to Fe and Mg, as well as Fe ions doping shows a higher charge transfer compared to Co doping. The photometric properties, i.e., CIE 1976 L^*^a^*^b^*^ coordinates, demonstrated that the doped TM ions as a chromophore not only enhanced the NIR reflectance but also enriched the hue of the pigments. The prepared pigments showcase exceptional stability against various chemicals, light, and temperature, rendering them highly suitable for preparing cool coatings. The energy‐saving simulations of the best‐performing Mg_2.95_Fe_0.15_TeO_6_ and Mg_2.95_Co_0.15_TeO_6_ pigments for cool coatings used in the arid desert climate of Riyadh can save 114 and 56 MJ/m^2^ energy, respectively, which is much higher than that of similar commercial cement and TiO_2_‐mica pearlescent pigment. Therefore, high NIR‐reflectance Mg_2.95_Fe_0.15_TeO_6_ and Mg_2.95_Co_0.15_TeO_6_ pigments could be used as promising cool coatings for sustainable solutions to alleviate heat accumulation due to sunlight and hence mitigate UHI.

## 4 Experimental Section

### 4.1 Materials Used

Magnesium dinitrate hexahydrate (Mg(NO_3_)_2_ · 6H_2_O; 99.99%), tellurium oxide (TeO_2_, 98%), ferric nitrate nonahydrate (Fe(NO_3_)_3_ · 9H_2_O, 98%), and cobalt (II) nitrate hexahydrate (Co(NO_3_)_2_ · 6H_2_O, 98%) were purchased from *Sigma–Aldrich Co*. A transparent synthetic varnish was procured from *Asian Paints Ltd*. for coating applications.

### 4.2 Synthesis of Mg_3−_ *_x_* A*_x_* TeO_6_ (A = Fe and Co; *x* = 0.05‐0.25 wt.%) Pigments

The colored Mg_3−_ *_x_* A*_x_* TeO_6_ (A = Fe and Co; *x* = 0.05–0.25 wt.%) pigments were synthesized via the SC route using laboratory‐prepared oxalyldihydrazide (ODH) as a fuel. A suitable quantity of TeO_2_ was placed in a petri dish and thoroughly dissolved in an aqueous NH_4_OH solution using a bath sonicator for 30 min. The resulting reaction mixture was kept on a hot plate with continuous stirring at a temperature of 90°C, producing the diammonium tellurate (H_8_N_2_O_4_Te) solution. The appropriate amount of Mg(NO_3_)_2_, Fe(NO_3_)_3_, H_8_N_2_O_4_Te, and ODH was taken in a petri dish and mixed in deionized water with continuous stirring for 10 min. The resultant solution was placed in a preheated muffle furnace set at 550°C. After just 10 min, the added fuel effectively causes the rapid disintegration of the oxidizers, leading to the release of gases such as CO_2_ and N_2_, along with a substantial amount of heat. The result of the combustion process was a fluffy powder featuring portholes. The significant energy released as heat during the combustion reaction played a crucial role in forming the highly crystalline structure of the synthesized pigment. The obtained powder was finely ground and calcined at 700°C for 3 h. The Mg_3−_ *_x_* Co*_x_* TeO_6_ (*x* = 0.05–0.25 wt.%) pigments were also synthesized by following the above synthesis protocol.

### 4.3 Characterizations

A Rigaku SmartLab diffractometer  was used for the XRD studies of the prepared pigments. The Rietveld refinements using the *Fullprof* suite program were used to study the crystal lattice parameters of the pigments. The morphology and elemental composition of the prepared pigments were analyzed using a Carl Zeiss, Germany‐made SEM EVO, MA‐10, coupled with EDX spectroscopy. TEM images were acquired using a JEOL‐made JEM 2100 Plus. A Bruker Alpha II‐ FTIR spectrometer with a diamond crystal attenuated total reflection (ATR) was used to study the functional groups/chemical bonds present in the pigments [35]. The XPS measurements were recorded on a Physical Electronics (PHI 5000 VersaProbe III) instrument. All binding energies were correlated to the peak at 283.7 eV belonging to the C 1s derived from adventitious carbon. The TCi thermal conductivity analyzer system (C‐Therm Technologies Ltd.) was employed to record the thermal conductivity of the cooling pigments. The UV—vis–NIR reflectance of the materials was recorded using a Perkin Elmer (Lambda 750) spectrophotometer in the spectral range 200–2500 nm. The solar reflectance of the cooling materials was estimated using the following equation;

(1) $$R^{*}=\frac{\int_{700}^{2500\;}r\left(\lambda \right)i\left(\lambda \right)d\lambda }{\int_{700}^{2500\;}i\left(\lambda \right)d\lambda }\;$$

here, r(*λ*): experimental reflectance data of the pigments, and i(λ): standard solar spectral irradiance as per ASTM standard model G173‐03. The IR emissivity data were attained from a Bruker Vertex 70v in the 1.25–28.25 µm wavelength range. The CIE 1976 L^*^a^*^b^*^ color parameters were documented using a Datacolor 800 benchtop dual beam d/8° spectrophotometer. Here, L^*^, a^*^, and b* represent the lightness scale (from 0 (black) to 100 (white)), green to red region, and blue to yellow region, respectively. The color saturation represented by C^*^ of the pigments was calculated using the equation given below [12]:

(2) $$C^{*}={\left[{\left(a^{*}\right)}^{2}+{\left(b^{*}\right)}^{2}\right]}^{1/2}\;$$

In addition, the *H°* (1‐360°) was calculated using the equation [12]:

(3) $$H^{\circ }=tan^{-1}\;\left(b^{*}/a^{*}\right)$$

A Philips BR125 IR 250 W bulb was used to study the photostability and thermal shielding of the pigments.

### 4.4 Interior Cooling Performance of the Prepared Pigments

The radiative cooling characteristic of the pigments was examined by a self‐made experimental setup. The best‐performing pigments were selected for NIR‐reflecting coatings over aluminum sheets. The procedure to fabricate the pigment coatings was as follows. The best‐performing Mg_2.85_Fe_0.15_TeO_6_ and Mg_2.85_Co_0.15_TeO_6_ pigments and synthetic varnish were taken in the weight ratio of 1:1 and thoroughly dispersed by using an ultrasound probe sonicator provided with a titanium horn for 1 h. The resultant‐colored emulsion was applied evenly to an aluminum sheet (10 × 10 cm^2^) with a paintbrush and permitted to dry at ambient temperature. Subsequently, coated aluminum sheets were roofed on the plywood house models (8 × 8 × 8 cm^3^). A small opening was created just 2 cm beneath the ceiling to allow for temperature measurement. The entire setup was then exposed to light from an IR lamp with a power density of 497 W/m^2^. At the same time, temperatures were logged every 5 min. The pictorial diagram represents the designed experiment setup to understand the cooling performance of the prepared pigments, as depicted in Figure S12.

### 4.5 Computational Details

We performed first‐principles DFT calculations using the Vienna Ab initio Simulation Package (VASP) [36, 37] to investigate the electronic and structural properties of pristine and doped Mg_3_TeO_6_. The interactions between ions and electrons were described using projector‐augmented wave (PAW) potentials [38, 39], while exchange‐correlation effects were treated within the generalized gradient approximation (GGA) using the Perdew–Burke–Ernzerhof (PBE) functional [40]. A plane‐wave basis set with an energy cutoff of 500 eV was employed to represent the Kohn–Sham orbitals. Structural optimization was carried out using the conjugate‐gradient algorithm until the Hellmann–Feynman forces on each atom were reduced below 0.005 eV·Å^−^^1^. To ensure convergence during geometry optimization, the Brillouin zone was sampled using a Γ‐centered Monkhorst–Pack k‐point mesh of 8 × 8 × 8. To analyze charge transfer, we employed Bader charge analysis, which calculates the net atomic charge using the relation: net charge = ZVAL − Bader population, where ZVAL represents the number of valence electrons for each atomic species [41, 42].

## Conflicts of Interest

The authors declare no conflicts of interest.

## Supporting information




**Supporting File**: smll73079‐sup‐0001‐SuppMat.docx.

## Data Availability

The data that support the findings of this study are available from the corresponding author upon reasonable request.
