# Supplementary material for: Site‐Engineered Corundum‐Structured Aesthetic Composites for Alleviating Heat Accumulation to Mitigate Urban Heat Islands
Source: Small. 2026 Mar 15;22(26):e13911. doi: 10.1002/smll.202513911 (PMC13155059; doi:10.1002/smll.202513911)
Supplement: Supplementary file 1 — Supporting File: smll73079‐sup‐0001‐SuppMat.docx. [file SMLL-22-e13911-s001.docx]

**Site-Engineered Corundum-Structured Aesthetic Composites for Alleviating Heat Accumulation to Mitigate Urban Heat Islands**

G.P. Darshan^1, 2 *^, Akshay Arjun^3^, Subhendu Mishra^4^, Abhishek Kumar Singh^4^,

Alberto Vomiero^5, 6*^, Elisa Moretti^5*^

*^1^Physics and Astronomy Department, University of Padova, Via Marzolo 8, Padova I-35131, Italy*

*^2^Department of Physics, Faculty of Natural Sciences, M S Ramaiah University of Applied Sciences, Ramaiah Technology Campus, Bengaluru 560058, India*

*^3^Department of Physics, RNS Institute of Technology, Bengaluru, 560 098, Karnataka, India*

*^4^Materials Research Centre, Indian Institute of Science, Bengaluru 560012, India*

*^5^Department of Molecular Sciences and Nanosystems, Ca’ Foscari University of Venice, Via Torino 155, Venezia, 30172, Italy*

*^6^Division of Materials Science, Department of Engineering Sciences and Mathematics, Luleå University of Technology, Luleå, 97187, Sweden*

**Keywords:** Urban heat islands; Chromophores; Photometric parameters; NIR reflectance

**Corresponding Authors:** Dr. Darshan G.P. (Email Id.: darshubavimane@gmail.com),

Prof. Alberto Vomiero (Email Id.: alberto.vomiero@ltu.se), and Dr. Elisa Moretti (Email Id.: elisa.moretti@unive.it)

Fig.S1. XRD patterns of the prepared pristine and Mg_3-_*_x_*Fe*_x_*TeO_6_ (*x* = 0.05, 0.1, 0.2, and 0.25 wt.%) pigments.

Fig.S2. XRD patterns of the prepared pristine, Mg_3-_*_x_*Co*_x_*TeO_6_ (*x* = 0.05, 0.1, 0.2, and 0.25 wt.%) pigments.

Fig.S3. FTIR spectra of the prepared pristine, Mg_2.85_Fe_0.15_TeO_6_, and Mg_2.85_Co_0.15_TeO_6_ pigments.


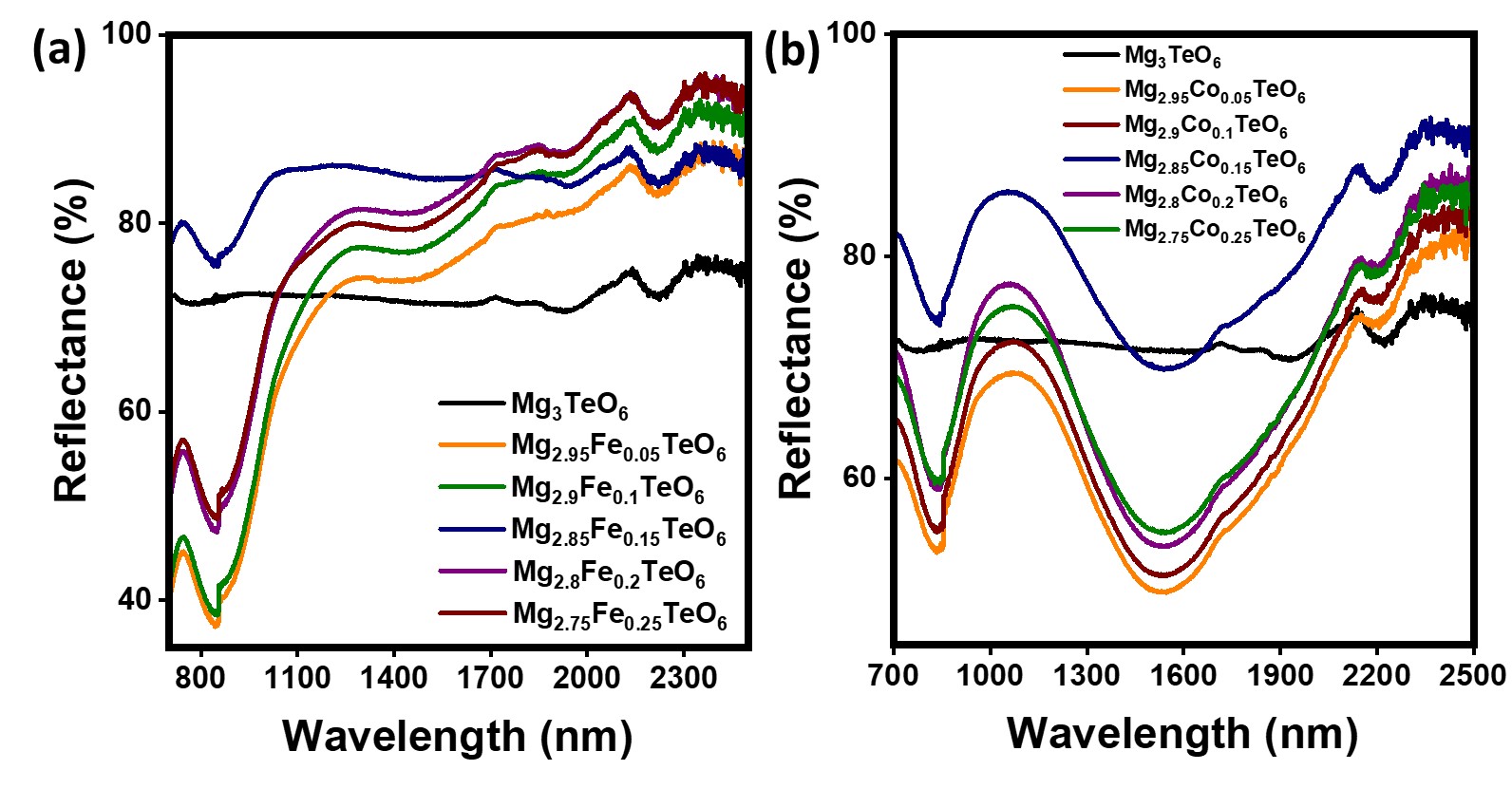


Fig. S4. NIR reflectance spectra of the prepared pigments from 700-2500 nm.


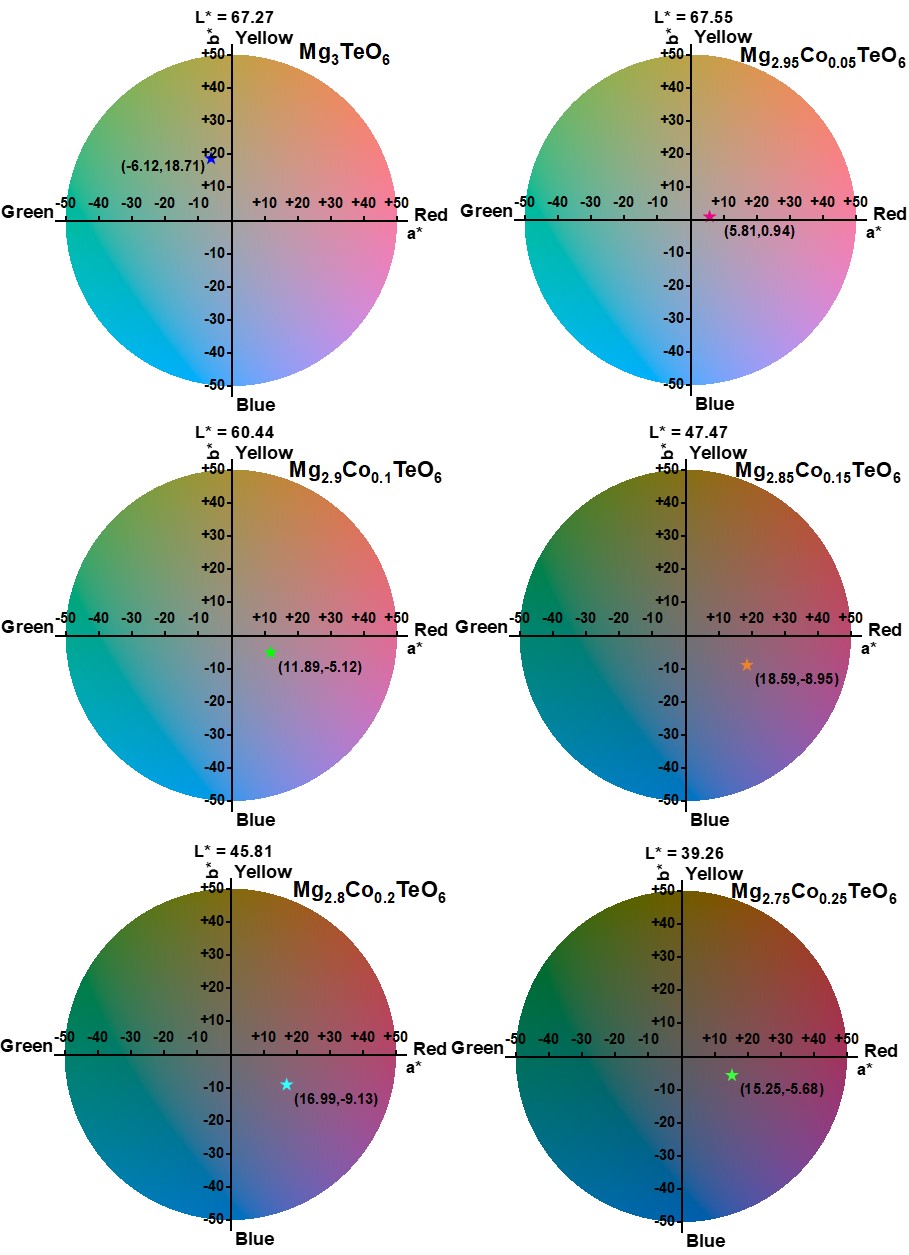


Fig.S5. CIE 1976 *L*a*b** diagrams of the prepared pristine and Mg_3-_*_x_*Co*_x_*TeO_6_ (*x*= 0, 0.05-0.25 wt. %) pigments.

Fig.S6. The IR emissivity spectra of prepared pristine and Mg_3-_*_x_*Fe*_x_*TeO_6_ (*x*= 0, 0.05-0.25 wt. %) pigments.

Fig.S7. The IR emissivity spectra of prepared pristine and Mg_3-_*_x_*Co*_x_*TeO_6_ (*x*= 0, 0.05-0.25 wt. %) pigments.


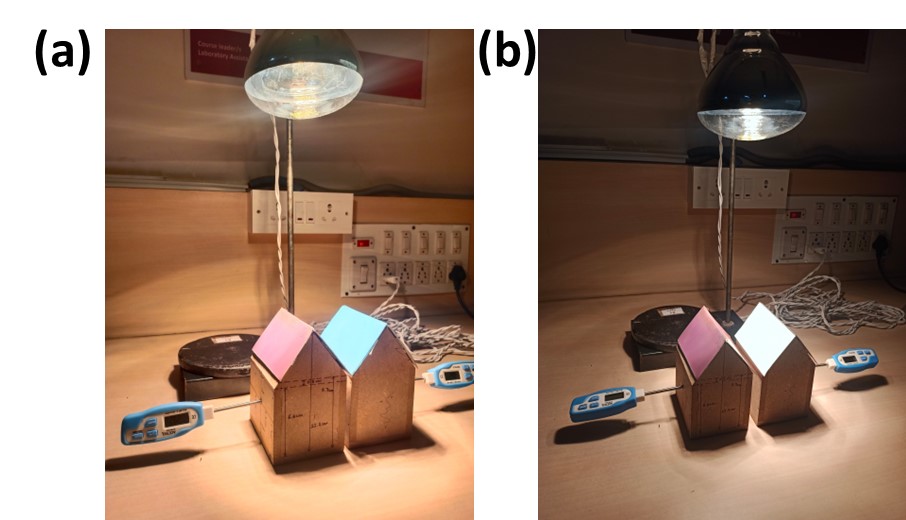


Fig.S8. Photographic images of the house models with different commercially available roofing materials were used to study passive daytime radiative cooling applications.

Fig.S9. The interior temperature of the designed house model with the best-performing Mg_2.95_Fe_0.15_TeO_6_-coated roofing and other traditional roofing materials.

Fig.S10. The interior cooling performance of Mg_2.95_Co_0.15_TeO_6_-coated roofing house model in comparison with other traditional roofing materials.


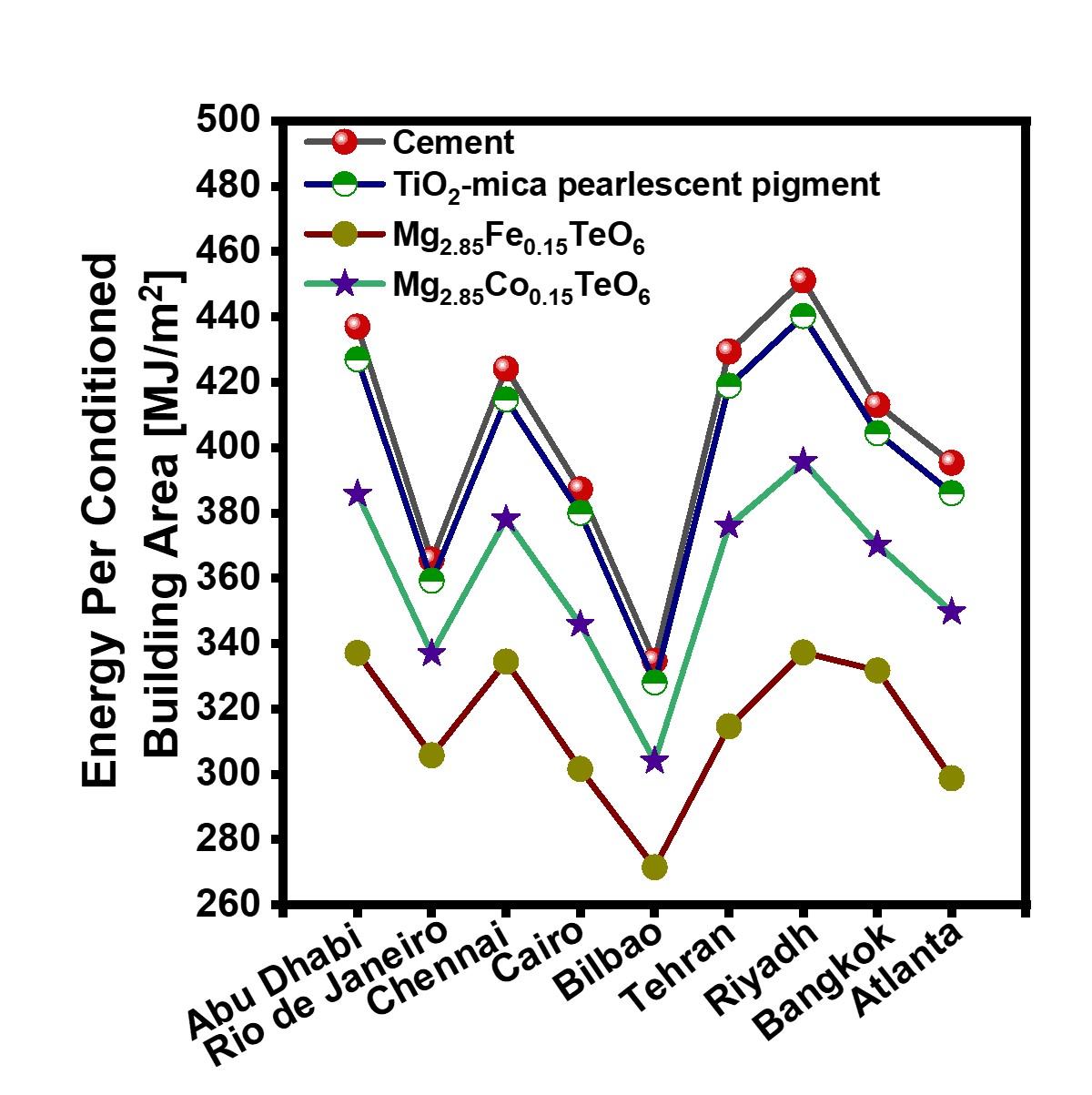


Fig.S11. Total energy demand per conditioned building area of cement, TiO_2_-mica pearlescent pigment, Mg_2.95_Fe_0.15_TeO_6_, and Mg_2.95_Co_0.15_TeO_6_ pigment coatings was simulated in the various regions with different climate zones.


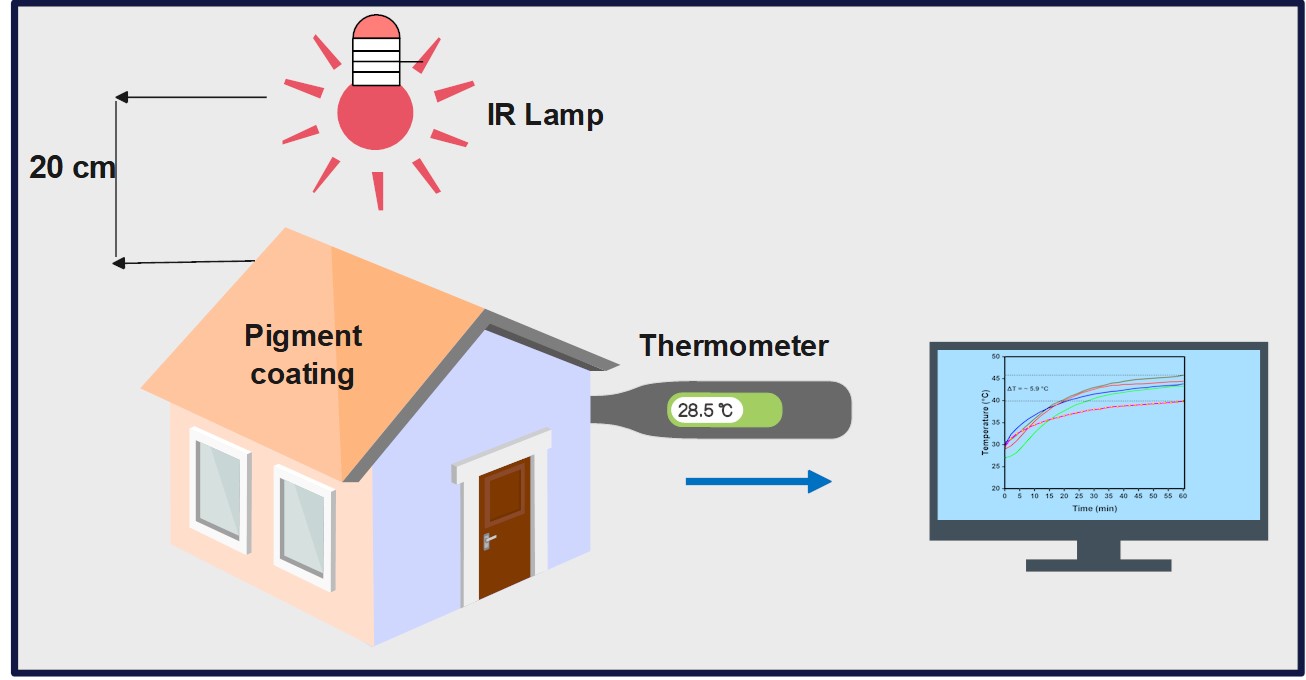


Fig.S12. The pictorial diagram represents the designed experiment setup to understand the cooling performance of the prepared pigments.

Table S1: List of previously reported NIR reflective pigments constituent of RE ions synthesized via various routes, and corresponding NIR reflectance values.

| **SI. No.** | **Pigments** | **Synthesis method** | **NIR reflectance (%)** | **External stimuli tests (Chemical/**  **Thermal/Photo)** | **References** |
| --- | --- | --- | --- | --- | --- |
| 01 | TbFeO_3_ | Coprecipitation | 48 | Yes | Maria et al. [1] |
| 02 | YFeO_3_ | Solid-state reaction | 45.8 | No | Li et al. [2] |
| 03 | La_1-_*_x_*Ce*_x_*AlO_3_ | Sol–gel | 82.22 | Yes | Cheng et al. [3] |
| 04 | LiCe(MoO_4+δ_)_2_ | Solid-state reaction | 95.28 | Yes | Xiao et al. [4] |
| 05 | YIn_0.9_*_x_*Mn_0.1_M*x*O_3−δ_ (M = Li/Zn, *x* = 0–0.4) | Solid-state reaction | 97.76 | Yes | Zhang et al. [5] |
| 06 | LiMgVO_4_:Co^2+^ and Ni^2+^ | Solid-state reaction | 82 | Yes | Maram et al. [6] |
| 07 | LiZnVO_4_:Co^2+^ | Solid-state reaction | 65.2 | No | Kshatri et al. [7] |

Table S2. The Rietveld refinement and reliability parameters of the pristine and best-performing pigments.

| **Parameters** | **Mg_3_TeO_6_** | **Mg_2.85_Fe_0.15_TeO_6_** | **Mg_2.85_Co_0.15_TeO_6_** |
| --- | --- | --- | --- |
| Crystal system | Hexagonal | | |
| Space group name | $\bar{R_{3}}$ | | |
| Space group number | 148 | | |
| a (Å) | 8.594 | 8.595 | 8.595 |
| b (Å) | 8.594 | 8.595 | 8.595 |
| c (Å) | 10.313 | 10.314 | 10.319 |
| α | 90° | 90° | 90° |
| β | 90° | 90° | 90° |
| γ | 120° | 120° | 120° |
| Unit cell volume (Å^3^) | 659.83 | 659.88 | 660.26 |
| R_p_ (%) | 2.73 | 2.55 | 2.79 |
| R_wp_ (%) | 3.50 | 3.27 | 3.58 |
| R_exp_ (%) | 3.17 | 3.04 | 3.60 |
| χ² (GoF) | 1.21 | 1.15 | 0.98 |
| R_Bragg_ (%) | 4.56 | 4.26 | 4.65 |
| R_F_ (%) | 7.64 | 7.14 | 7.81 |

Table S3. The NIR reflectance of various Fe and Co-doped pigments has been reported in previous literature.

| SI. No. | Pigments | Synthesis method | Structure | NIR reflectance (%) | CIE values | References |
| --- | --- | --- | --- | --- | --- | --- |
| 01 | BaSn_1–_*_x_*Fe_x_O_3−δ_ | Sol–gel | Cubic | 79.81 | *L** = 63.70  *a** = 5.78  *b** = 15.74 | Jieyu Wang et al. [8] |
| 02 | ZnAl_2-_*_y_*Fe*_y_*O_4_ (y = 0–0.5) | Citrate sol-gel | Cubic spinel | 81 | *L** = 87.49  *a** = 2.00  *b** = 16.53 | Elakkiya et al. [9] |
| 03 | Co_0.5_Mg_0.5_Al_2 −_*_x_*Fe*_x_*O_4_ | Pechini-type sol–gel | Spinel | 52.7 | *L** = 49.79  *a** = − 16.09  *b** = − 10.48 | Weiwei et al. [10] |
| 04 | YAl_1−_*_x_*Fe*_x_*O_3_ | Modified Pechini | Orthorhombic | 70 | *L** = 78.01  *a** = -2.46  *b** = 26.10 | Silvia et al. [11] |
| 05 | Mg_2.85_Fe_0.15_TeO_6_ | Solution combustion | Hexagonal | 86 | *L** = 51.43  *a** = 15.91  *b** = 14.85 | Present work |
| Co-doped pigments | | | | | | |
| 01 | Ca_1-_*_x_*La*_x_*Al_12-_*_x_*Co*_x_*O_19_ (x = 0–0.5) | Combustion | Hibonite | 70.7 | *L** = 73.3  *a** = −2.6  *b** = −34.1 | Rus et al. [12] |
| 02 | BaAl_1.6_Co_0.4_Si_2_O_8_ | Solid-state | Monoclinic | -- | *L** = 62.96  *a** = 6.05  *b** = 27.03 | Wang et al. [13] |
| 03 | Zn_0.5_Co_0.5_Fe_2_O_4_ | Solid-state | Cubic spinel | 38 | *L** = 26.89  *a** = 2.91  *b** = 2.34 | Sameera et al. [14] |
| 04 | NaZn_0.9_Co_0.025_PO_4_ | Solid-state | Monoclinic | 67 | *L** = 60.43  *a** = 3.68  *b** = −41.66 | Thejus et al. [15] |
| 05 | Mg_2.85_Co_0.15_TeO_6_ | Solution combustion | Hexagonal | 86 | *L** = 47.47  *a** = 18.59  *b** = -8.95 | Present work |

Table S4. CIE 1976 *L*a*b**, *C**, and *H°* values of the prepared pigments.

| **Materials** | ***L*** | ***a**** | ***b**** | ***C**** | ***Hº*** |
| --- | --- | --- | --- | --- | --- |
| Mg_3_TeO_6_ | 67.27 | -6.12 | 18.71 | 19.69 | 108.11 |
| Mg_2.95_Fe_0.05_TeO_6_ | 68.60 | 12.99 | 20.52 | 24.29 | 57.66 |
| Mg_2.9_Fe_0.1_TeO_6_ | 54.18 | 6.09 | 17.94 | 18.95 | 71.25 |
| Mg_2.85_Fe_0.15_TeO_6_ | 51.43 | 15.91 | 14.85 | 21.76 | 43.03 |
| Mg_2.8_Fe_0.2_TeO_6_ | 45.98 | 13.90 | 11.71 | 18.18 | 40.11 |
| Mg_2.75_Fe_0.25_TeO_6_ | 38.31 | 14.24 | 16.00 | 21.42 | 48.33 |
|  |  |  |  |  |  |
| Mg_2.95_Co_0.05_TeO_6_ | 67.55 | 5.81 | 0.94 | 5.89 | 9.19 |
| Mg_2.9_Co_0.1_TeO_6_ | 60.44 | 11.89 | -5.12 | 12.95 | 336.70 |
| Mg_2.85_Co_0.15_TeO_6_ | 47.47 | 18.59 | -8.95 | 20.63 | 334.29 |
| Mg_2.8_Co_0.2_TeO_6_ | 45.81 | 16.99 | -9.13 | 19.29 | 331.75 |
| Mg_2.75_Co_0.25_TeO_6_ | 39.26 | 15.25 | -5.68 | 16.27 | 339.57 |

Table S5. The climate information of the selected cities for energy simulation.

| **ASHRAE climate zones** | **Climate type** | **Country** | **City** | **Reason for selecting** |
| --- | --- | --- | --- | --- |
| **1A** | Very Hot – Humid | 🇮🇳 **India** | **Chennai** | Hot and humid, year-round; excellent for coastal and tropical zone simulation |
| **1B** | Very Hot – Dry | 🇸🇦 **Saudi Arabia** | **Riyadh** | High solar intensity, arid desert climate |
| **2A** | Hot – Humid | 🇹🇭 **Thailand** | **Bangkok** | Tropical monsoon climate, consistently hot and humid, ideal for Southeast Asian studies |
| **2B** | Hot – Dry | 🇦🇪 **UAE** | **Abu Dhabi** | Very high solar radiation, reflective surfaces most effective |
| **3A** | Warm – Humid | 🇧🇷 **Brazil** | **Rio de Janeiro** | Urban coastal heat, moderate-high humidity |
| **3B** | Warm – Dry | 🇪🇬 **Egypt** | **Cairo** | Intense sun, moderate winters, excellent to test pigment performance |
| **3C** | Warm – Marine | 🇪🇸 **Spain** | **Bilbao** | Mild coastal climate; fluctuating humidity, useful for marine exposure testing |
| **4A** | Mixed – Humid | 🇺🇸 **USA** | **Atlanta** | For extended analysis on seasonal effects of coatings |
| **4B** | Mixed – Dry | 🇮🇷 **Iran** | **Tehran** | Good for semi-arid zone if extending beyond hot climates |

**References:**

1. M. Fortuño-Morte, P. Serna-Gallén, H. Beltrán-Mir, E. Cordoncillo, *J. Materiomics,* 2021, **7**, 1061-1073.
2. Y. Li, Y. Ma, W. liu, Z. Wang, H. Liu, X. Wang, H. Wei, S. Zeng, N. Yi, G.J. Cheng, *Sol. Energy*, 2021, **226**, 180-191.
3. C. Chen, A. Han, J. Wang, Y. Su, M. Ye, *Sol. Energy*, 2023, **262**, 111857.
4. Y. Xiao, L. Feng, B. Huang, J. Chen, W. Xie, X. Sun, *Ceram. Int.* 2021, **47**, 29856-29863.
5. M. Zhang, L. Feng, Z. Zeng, Y. Yang, X. Sun, *ACS Sustain. Chem. Eng.* 2022, **10**, 13877-13886.
6. Durga Lalitha Bai Kshatri, Varadaraju Upadhyayula Venkata, Pardha Saradhi Maram, *Inorg. Chem*. 2025, **64, 50**, 25015-25024.
7. Durga Lalitha Bai Kshatri, Varadaraju Upadhyayula, Surfarazhussain Halkarni, Ch Subrahmanyam, Pardha Saradhi Maram, *Adv. Funct. Mater.* 2025, e21942.
8. Jieyu Wang, Aijun Han, Mingquan Ye, Chenlu Chen, Xin Chen, Xufei Zhu, *ACS Sustainable Chem. Eng.* 2021, **9, 48**, 16328-16337.
9. V. Elakkiya, Shanmugam Sumathi, *J. Alloys Compd.* 2020, **820**, 153174.
10. Weiwei Bao, Fei Ma, Yingtang Zhang, Xiaoli Hao, Zhifeng Deng, Xiangyu Zou, Wei Gao, *Powder Tech.* 2016, **292**, 7-13.
11. Silvia Blasco-Zarzoso, Héctor Beltrán-Mir, Eloísa Cordoncillo, *J. Alloys Compd.* 2023, **960**, 170695.
12. I. Rus, R. Ianoș, R. Lazău, C. Păcurariu, *Mat. Today Chem*. 2023, **28**, 101391.
13. Zhiwei Wang, Qiuying Wang, Qin Liu, Suwit Suthirakun Wongsathorn Kaewraung, Peng Jiang, Hang Zhao, Xin Xin, M. A. Subramanian, *ACS Appl. Opt. Mater.* 2024, **2, 2**, 313-322.
14. Saithathul Fathima Sameera, Ancy Mini Vibin Lal Nayakom Mini, Muhammed Fayis Athyaparambath, Mohammed Aysha Shafna, Sheik Muhammadhu Aboobakar Shibli, *ACS Appl. Eng. Mater.* 2023, **1, 10**, 2510-2523.
15. P.K. Thejus, K.V. Krishnapriya, K.G. Nishanth, *Sol. Energy Mater. Sol. Cell*. 2021, **219**, 110778.
